# One-pot universal NicE-seq: all enzymatic downstream processing of 4% formaldehyde crosslinked cells for chromatin accessibility genomics

**DOI:** 10.1186/s13072-021-00427-2

**Published:** 2021-12-11

**Authors:** Udayakumar S. Vishnu, Pierre-Olivier Estève, Hang Gyeong Chin, Sriharsa Pradhan

**Affiliations:** Genome Biology Division, New England Biolabs, Inc., Ipswich, MA 01983 USA

**Keywords:** Accessibility, Chromatin, Crosslinking, DNA-damage, Fixed-cells, NicE-seq

## Abstract

**Background:**

Accessible chromatin landscape allows binding of transcription factors, and remodeling of promoter and enhancer elements during development. Chromatin accessibility along with integrated multiomics approaches have been used for determining molecular subtypes of cancer in patient samples.

**Results:**

One-pot Universal NicE-seq (One-pot UniNicE-seq) is an improved accessible chromatin profiling method that negate DNA purification and incorporate sonication free enzymatic fragmentation before library preparation and is suited to a variety of mammalian cells. One-pot UniNicE-seq is versatile, capable of profiling 4% formaldehyde fixed chromatin in as low as 25 fixed cells. Accessible chromatin profile is more efficient on formaldehyde-fixed cells using one-pot UniNicE-seq compared to Tn5 transposon mediated methods, demonstrating its versatility.

**Conclusion:**

One-pot UniNicE-seq allows the entire process of accessible chromatin labeling and enrichment in one pot at 4% formaldehyde cross-linking conditions. It doesn’t require enzyme titration, compared to other technologies, since accessible chromatin is labelled with 5mC incorporation and deter degradation by nicking enzyme, thus opening the possibility for automation.

**Supplementary Information:**

The online version contains supplementary material available at 10.1186/s13072-021-00427-2.

## Background

Eukaryotic nuclear DNA is packaged into chromatin with histone and non-histone proteins, including reader, writer, eraser, and other chromatin-associated proteins [1]. The cumulative influence of these protein factors determines the transcriptional state of the chromatin [2]. Upon biological and environmental signals, nuclear chromatin undergoes remodeling, providing accessibility to DNA-binding proteins including transcription factors to initiate gene expression. Gene expression is dynamic, and often involves chromatin changes involving both DNA methylation and post-translational histone modification [3–6]. Furthermore, at the transcription start site, the chromatin is accessible for transcription preinitiation complex assembly for RNA synthesis. Although housekeeping genes are generally accessible at their promoter throughout development, tissue and stage-specific gene expression are regulated via chromatin accessibility and other cis interacting elements. In a comprehensive study, the chromatin accessibility landscape is gradually established during early human embryogenesis and was distinctive from mice [7]. To understand how these processes occur in vivo, chromatin accessibility snapshot would be of immense help. Indeed, in a landmark study using genome-wide chromatin accessibility profiles of 410 tumor samples representing 23 cancer types from The Cancer Genome Atlas [8], Cheng and Greenleaf’s laboratories have identified 562,709 transposase-accessible DNA elements that substantially extended the number of known cis-regulatory elements. Further analysis and integration of these accessible element data sets with TCGA multi-omic data identified a large number of putative distal enhancers that distinguished molecular subtypes of cancers. These data further identified putative genetic risk loci for cancer predisposition as active DNA regulatory elements in the cancer genome. It also identified the gene-regulatory network underlying cancer immune evasion, and further pinpointed noncoding mutations that drive enhancer activation, and perhaps its impact on patient survival [9]. Similar data sets analysis of TCGA archived cancer patient chromatin accessibility data sets also demonstrated striking chromatin accessibility difference between lung adenocarcinoma and lung squamous cell carcinoma, and between basal and non-basal breast cancer [10].

Chromatin accessibility studies have utilized a variety of protocols using formaldehyde as a fixative agent, including Formaldehyde-Assisted Isolation of Regulatory Elements (FAIRE-seq) [11]; DNase hypersensitive region sequencing (DNase-seq) [12]; Assay for Transposase-Accessible Chromatin sequencing (ATAC-seq) [13]; and more recently, Nicking Enzyme assisted sequencing (NicE-seq) [14]. All the above methods work in optimal sample preparation to a varying degree, each one needs some protocol optimization based on sample type, fixation condition and amounts of experimental materials. While ATAC-seq works primarily on unfixed cells, mitochondrial DNA sequence contamination was a major issue until a modified Omni-ATAC-seq protocol was developed [15]. Similarly, NicE-seq needed optimization of nicking enzyme concentration to cell number in the reaction, until universal NicE-seq was implemented using a 5mdCTP during polymerase mediated accessible region extension. The addition of 5mdCTP prevented further nicking and degradation of accessible chromatin DNA. Universal NicE-seq typically uses formaldehyde to cross-link chromatin, and an undeniably powerful approach for determining chromatin accessibility in culture cells, frozen tissue sections, and FFPE sections with relatively intact DNA [16, 17].

Formaldehyde is the most used aldehyde to fix cells and tissues. Being an electrophilic molecule, it is susceptible to chemical attack by a wide range of nucleophilic species of biological interest and is widely used in biological experiments, such as chromatin immunoprecipitation sequencing (ChIP-seq), DNase hypersensitive site sequencing (DNase-seq), and various other in situ labeling and hybridization experiments. It has been known that the crosslinked products can include intramolecular and intermolecular crosslinked species that would strongly influence the nature, yield, and half-life of chemical modifications [18]. Furthermore, the concentration of formaldehyde used, incubation times, and other conditions, including temperature of the reaction can have a profound impact on the fixation reaction, resulting in a wide array of chemical adducts [19]. Formaldehyde reacts with amino and imino groups of DNA bases, and extensive studies have been performed to document the kinetic and thermodynamic aspects of the reactions [20–24]. Indeed, formaldehyde reactivity with DNA is notably different as covalent modification of DNA bases requires disruption of base pairing in duplex DNA. Formaldehyde mainly induces N-hydroxymethyl mono-adducts on guanine, adenine and cytosine, and N-methylene crosslinks between adjacent purines in DNA. This leads to DNA damage and strand break that would affect DNA polymerization reaction.

Indeed, there is a lack of comprehensive studies of various fixing conditions on accessible chromatin analysis. Since universal NicE-seq relies on sequence-specific nicking of the DNA strand, followed by nick translation using DNA polymerase I on formaldehyde-fixed cells to reveal accessible chromatin following library sequencing, the residual nicks or DNA damages may be adverse. To study these processes, we first measured the DNA damage markers on formaldehyde fixed cells and subsequently developed a genome-wide accessible chromatin library method that would not require DNA purification or sonication steps for fragmentation. Indeed, from the experimental samples to library preparation can be performed in one tube. Using this novel method, we performed detailed chromatin accessibility studies in four different cell lines and compared them with formaldehyde-fixed ATAC-seq and scATAC-seq.

## Results

### DNA damage pathway activation following formaldehyde fixation

**Fig. 1:**
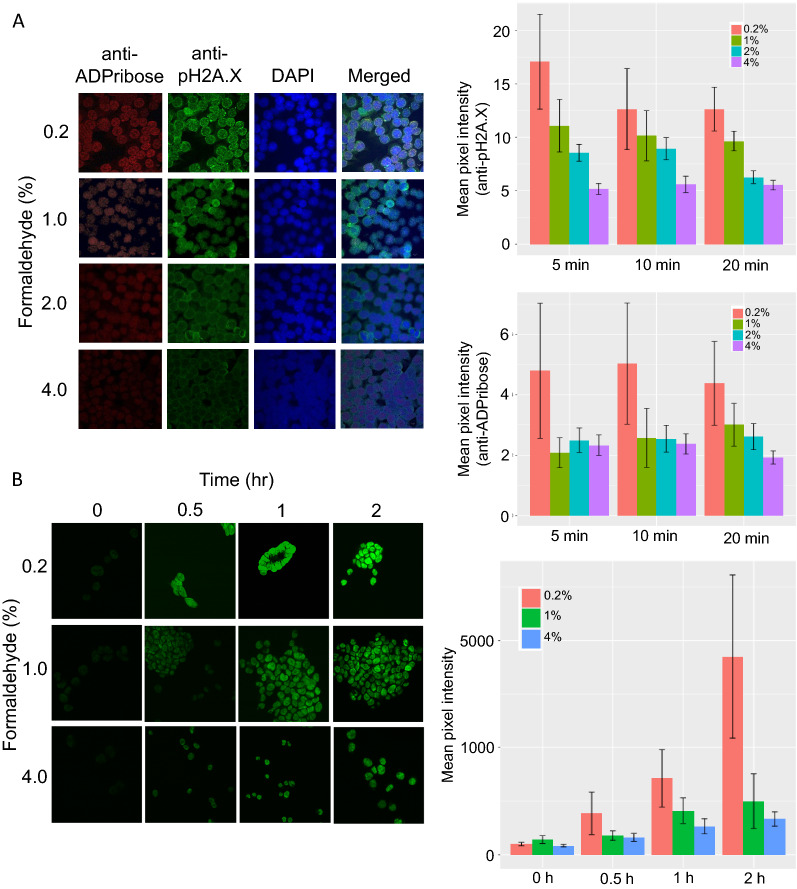
Formaldehyde fixation condition, DNA damage response pathways markers and one-pot UniNicE-seq labeling efficiency in HCT116 cells. **A** Left panel, representative visualization of ADPribose and phosphoSer139 histone H2A.X using 561 nm (red) and 488 nm (green) laser of cells fixed with different concentration of formaldehyde as indicated in Y axis. The antibodies used for probing are indicated on top. DNA content of nuclei is revealed by DAPI (blue) staining. Right panel shows quantification of fluorescence, represented as mean pixel intensity from anti-pH2A.X and anti-ADPribose staining. **B** Visualization of NicE-view using fluorescein-dATP (green) on cells differently fixed with different formaldehyde concentrations (Y-axis) at different time intervals indicated on the top. Left panel shows Fluorescein-12-dATP labeling in a one-pot UniNicE-seq reaction. The labeling efficiency is shown at right panel

When mammalian cells are exposed to genotoxic stress, the DNA damage pathway is activated. This results in poly ADP-ribosylation of proteins at DNA damage sites, and occupancy of phospho-H2A.X (pH2A.X) at the damaged site. Since formaldehyde is a potent genotoxic stress agent used for fixation of cells prior to accessible chromatin analysis, we investigated if DNA damage pathways are activated during formaldehyde cell fixation process. Anti-pH2A.X and anti-PolyADP-ribose antibodies were used in immunocytochemistry to visualize and quantitate the presence of these two marks in the nucleus. We used a series of formaldehyde concentrations ranging between 0.2 and 4% to fix HCT116 cells for 5, 10 and 20 min, and measured the accumulation of both pH2A.X foci and poly ADP-ribosylation. Indeed, the highest accumulation of both pH2A.X and poly ADP-ribosylation was observed at 0.2% formaldehyde fixation irrespective of fixing time for HCT116 cells (Fig. 1A). However, pH2A.X and poly ADP-ribosylation signals dropped as the concentration of formaldehyde was raised to 1% and remained stable at 4% fixation conditions. At 4% formaldehyde, 5 min fixation time didn’t generate additional pH2A.X and poly ADP-ribosylation signals and was comparable to longer 10- or 20-min fixation.

### Formaldehyde fixation condition and accessible chromatin labeling efficiency

Since universal NicE-seq uses a nicking enzyme Nt.CviPII to label accessible chromatin DNA on cells fixed with formaldehyde, we pursue our studies of NicE-seq labeling efficiency in various formaldehyde fixing conditions. We investigated if newly generated polyADP-ribosylation and pH2A.X on chromatin during fixation may affect accessible chromatin labeling. For this work HCT116 cells fixed with 0.2, 1, and 4% formaldehyde were subjected to universal NicE-seq labeling reaction using fluorescein conjugated dATP in the nucleotide mixture for 0–2 h. We monitored the labeling efficiency by fluorescein-incorporation measurement of the nucleus. As expected, at time 0, there were no fluorescein labelled nuclei, and the labeling increased up to two hrs. However, to our surprise, we observed intense fluorescein labeling at 0.2% formaldehyde fixation despite high poly-ADP ribosylation and pH2A.X accumulation of the nuclei (Fig. 1B). We also observed fixed cells displayed more clumping at 0.2% formaldehyde compared to 1 or 4% (data not shown). Although, the nuclear staining using DAPI displayed lower pixel intensity in 0.2% compared to 1 or 4% formaldehyde fixation condition. We hypothesized that 0.2% formaldehyde fixation may result in poor fixing of the cellular components resulting in random nicks and greater polymerase mediated fluorescein-conjugated dATP incorporation or altered nuclear structure resulting in strong fluorophore incorporation compared to DAPI.

### Enzymatic method of accessible chromatin library at 4% formaldehyde fixed cells

**Fig. 2:**
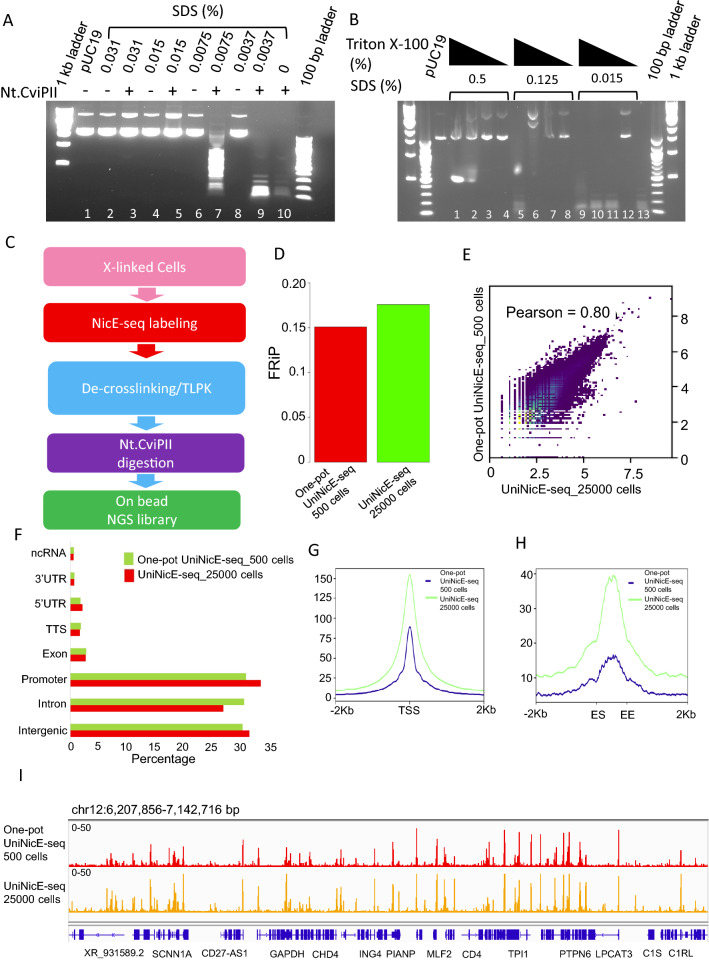
Optimization of sonication free enzymatic condition for UniNicE-seq. **A** Effect of SDS concentrations in Nt.CviPII activity on plasmid DNA. Lane number 10 shows complete digestion of the plasmid DNA in the absence of SDS. **B** Effect of Triton X-100 in quenching of SDS mediated inhibitory activity of Nt.CviPII. Lane number 13 is the positive control. Triton X-100 concentrations were 1, 0.5, 0.125 and 0.1%, respectively. **C** Schematic representation of one-pot universal NicE-seq. **D** Comparison of FRiP scores of one-pot UniNicE-seq 500 cells and UniNicE-seq 25,000 cells. **E** Genome-wide comparison of accessible chromatin between one-pot UniNicE-seq 500 cells and UniNicE-seq 25,000 cells using sequence read density. **F** Peak annotation of one-pot UniNicE-seq 500 cells and UniNicE-seq 25,000 cells. **G** Genome-wide metagene plot of transcription start site (TSS) with ± 2 Kb of flanking region of one-pot UniNicE-seq 500 cells and UniNicE-seq 25,000 cells. **H** Genome-wide metagene plot of enhancer elements with ± 2 Kb of flanking region following enhancer start (ES) and enhancer end (EE) site of one-pot UniNicE-seq 500 cells and UniNicE-seq 25,000 cells. **I** Representative IGV genomic tracks of accessible chromatin of one-pot UniNicE-seq 500 cells compared with UniNicE-seq 25,000 cells. Gene names are indicated at the bottom

Robust 4% formaldehyde fixing condition doesn’t allow poly-ADP ribosylation or pH2A.X accumulation in cells and is often used in clinical laboratories for tissue fixation. Therefore, we developed a robust accessible chromatin protocol using HCT116 as a model cell line. We modified the universal NicE-seq protocol that routinely used 1% formaldehyde fixation and applied it to 4% formaldehyde fixation and tested its efficiency in different cell numbers. Accessible chromatin analysis in low cell numbers, particularly < 1000 is challenging for isolation of the genomic DNA. We, therefore, modified the protocol to negate DNA isolation and sonication steps before NGS library preparation. In the universal NicE-seq protocol, the labeling reaction allows the incorporation of 5mdCTP into the accessible regions. The presence of 5mdCTP in these regions confer resistant to repeated nicking and degradation of it by Nt.CviPII. We hypothesized that a second incubation of Nt.CviPII would allow nicking of chromatin DNA other than the 5mC incorporated accessible regions post-decrosslinking and proteinase K treatment. However, the decrosslinking reaction contained 0.8% SDS that would render the nicking enzyme catalytically inactive. Therefore, we tittered the SDS concentration that would allow the nicking enzyme to remain catalytically active. In this experiment, we incubated pUC19 DNA with various concentrations of SDS and added Nt.CviPII to observe its activity by analyzing the digested products on a agarose gel. Indeed, SDS was a strong inhibitor of Nt.CviPII, till 0.008% concentration (Fig. 2A). Since SDS is crucial for protein denaturation that aids in proteinase K activity, it can’t be completely removed from the reaction. This led us to investigate if we can use a quencher for SDS, such as NP40, Triton X-100, or sodium deoxycholate that would allow the Nt.CviPII activity in the 2nd reaction. Indeed, SDS inhibition of Nt.CviPII was effectively quenched by the addition of Triton X-100 (Fig. 2B). At 0.015% SDS in the reaction, a tenfold Triton X-100 mix was found to be ideal for Nt.CviPII activity.

The deproteination of the crosslinked DNA by proteases requires higher SDS concentration; therefore, we used 10 folds excess SDS (0.15%) in the presence of thermolabile proteinase K (TLPK). After the deproteination reaction, TLPK was heat-inactivated at 55 °C for 10 min and the reaction was diluted 10 folds and adjusted with Triton X-100 before 2nd Nt.CviPII digestion. We term this new method as one-pot UniNicE-seq (Fig. 2C). To test this new sonication-free one-pot UniNicE-seq enzymatic method, we used 500 HCT116 cells fixed with 4% formaldehyde and made accessible chromatin library on beads in duplicates and compared with previously published HCT116 accessible chromatin library using purified DNA from 25,000 labeled cells. Indeed, both libraries had comparable FRiP scores (Fig. 2D). The Pearson’s correlation between these libraries were r = 0.8 with similar genomic features, TSS, enhancer, and IGV profile (Fig. 2E–I). Taken together, we concluded that enzymatic accessible libraries are comparable between both methods. It would also allow library making without DNA purification and sonication, thus amenable to possible automation in the future.

### Formaldehyde fixing conditions affects chromatin accessibility

To carefully determine the effect of cell fixing in different formaldehyde conditions, we chose 0.2, 1 and 4% formaldehyde as fixative prior to NicE-seq labeling and made accessible chromatin libraries in duplicate using HCT116 cells and sequenced the library in depth. First, we measured the fraction of reads in peak (FRiP) to determine the quality of libraries in downsized sample representing similar read numbers. The FRiP score for 1 and 4% formaldehyde fixed libraries were ~ 0.19 compared to ~ 0.08 for 0.2% fixed library (Additional file 1: Fig. S1A). Although the Pearson’s correlation analysis was comparable between all three conditions ( r = 0.73–1.0), the TSS profile that represents the bulk of the accessible region and a good qualitative matrix, was weakly enriched in 0.2% formaldehyde fixed cells compared to 1 or 4% fixed cells (Additional file 1: Fig. S1B, C). On a closer inspection of the IGV track, we also observed that the signal-to-noise ratios for 1 and 4% formaldehyde fixed cells were higher compared to 0.2%, confirming poor fixing conditions results in non-specific nicks throughout, perhaps contributing to the noise (Additional file 1: Fig. S1D). This result correlates with our previous observation of higher staining intensity in 0.2% fixation library due to non-specific labeling (Fig. 1B). It also confirms that formaldehyde fixing condition at or above 1% would be ideal for genome-wide accessible chromatin analysis, since there was no significant difference in TSS heat map or IGV signal between 1 and 4% fixed cells (Additional file 1: Fig. S1C, D).

### Enzymatic method of accessible chromatin library with varied cell numbers

**Fig. 3:**
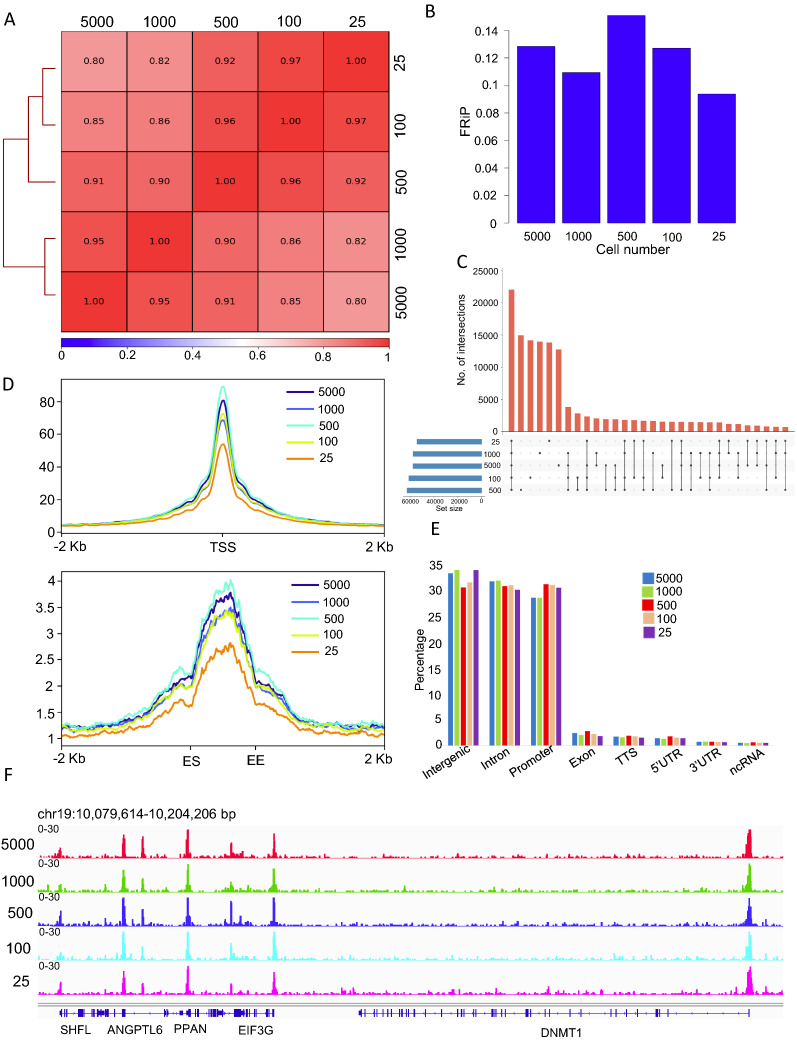
Sonication free enzymatic condition for accessible chromatin from 25 to 5 K HCT116 cells. **A** Pearson correlation of accessible chromatin peak read densities between one-pot UniNicE-seq 5000, 1000, 500, 100 and 25 cells. **B** Comparison of FRiP scores of one-pot UniNicE-seq 5000, 1000, 500, 100 and 25 cells. **C** Upset plot showing common and unique accessible region peaks between one-pot UniNicE-seq 5000, 1000, 500, 100 and 25 cells. **D** Genome-wide metagene plot of TSS (top panel) and enhancer elements (bottom panel) with ± 2 Kb of flanking region of one-pot UniNicE-seq 5000, 1000, 500, 100 and 25 cells. Enhancer start (ES) and enhancer end (EE) site are indicated at the bottom. **E** Peak annotation of one-pot UniNicE-seq 5000, 1000, 500, 100 and 25 cells libraries. **F** Representative IGV genomic tracks of accessible chromatin of one-pot UniNicE-seq 5000, 1000, 500, 100 and 25 cells

Since the majority of clinical samples at fixed with 4% formaldehyde and this process also inhibited poly ADP-ribosylation during the fixing process, we performed one-pot UniNicE-seq using the enzymatic method for accessible chromatin. We used four different cell lines, HCT116, HeLa, HEK293 and GM12878 to establish general applicability, and to investigate limitation of our new method. We used cell numbers varied from 5000, 1000, 500, 100 and 25 and made accessible chromatin library in replicates and performed analysis. The replicates for HCT116 cells were compared for FRiP, Pearson correlation, and reproducibility of TSS ± 2 Kb profile (Additional file 1: Fig. S2). The FRiP scores for replicates were reproducible (5 K cells, r = 0.187 and 0.188, 1 K cells, r = 0.158 and 0.16, 500 cells, r = 0.186 and 0.19, 100 cells, r = 0.136 and 0.142, 25 cells, r = 0.075 and 0.091., Additional file 1: Fig. S2A). Similarly, the Pearson correlation between replicates of 25–5 K cells was consistent and above r = 0.94 (Additional file 1: Fig. S2B–F). The TSS heat maps between replicates were almost identical (Additional file 1: Fig. S2G). Taken together, we demonstrated that one-pot UniNicE-seq is technically reproducible. We next merged the replicates for each cell line and performed analysis. The Pearson’s correlations between libraries for HCT116 ( r = 0.8–0.97; Fig. 3A), HeLa ( r = 0.94–0.98; Additional file 1: Fig. S3A), HEK293 ( r = 0.95–0.99; Additional file 1: Fig. S4A) and GM12878 ( r = 0.76–0.95; Additional file 1: Fig. S5A) suggesting good correlation between libraries despite variable cell numbers. The FRiP scores varied between 0.09 and 0.15 for HCT116, indicating reliability in peak identification in NGS analysis (Fig. 3B). The upset plot of overlapping accessible chromatin peaks between different cell numbers also demonstrated the majority of the peaks (> 50%) are common amongst them for HCT116 cells (Fig. 3C). Indeed, the common peaks between 25 and 5 K cells were 50% for HCT116, 70% for HeLa, 75% for HEK293 and 65% for GM12878 cells (Fig. 3C, Additional file 1: Figs. S3B, S4B, S5B). As expected, the accessible regions of the chromatin were enriched at the TSS and enhancer (Fig. 3D; Additional file 1: Figs. S3C, S4C, S5C). The distribution of genomic features between 25 and 5000 cells remained consistent (Fig. 3E; Additional file 1: Figs. S3D, S4D, S5D). Upon inspection of IGV tracks, it was apparent that the signal-to-noise ratios of accessible chromatin peaks between different numbers of cells was similar for all the four cell lines used in our validation (Fig. 3F, Additional file 1: Figs. S3E, S4E, and S5E). However, as expected, the reduction of cell numbers resulted in a loss of accessible chromatin peak. To determine the genomic distribution of the non-overlapping peaks in HCT116 data sets comprising of 5 K, 1 K, 500, 100 and 25 cells, we performed peak annotation (Additional file 1: Fig. S6). Indeed, loss of accessible peaks was evenly distributed across genomic features, suggesting cell number increase contributed to additional accessible chromatin. These results demonstrate robust accessible chromatin profiling at low cell numbers and the method could be adapted universally.

### Genomic and epigenomic accessible chromatin features are maintained in 25 cells

**Fig. 4:**
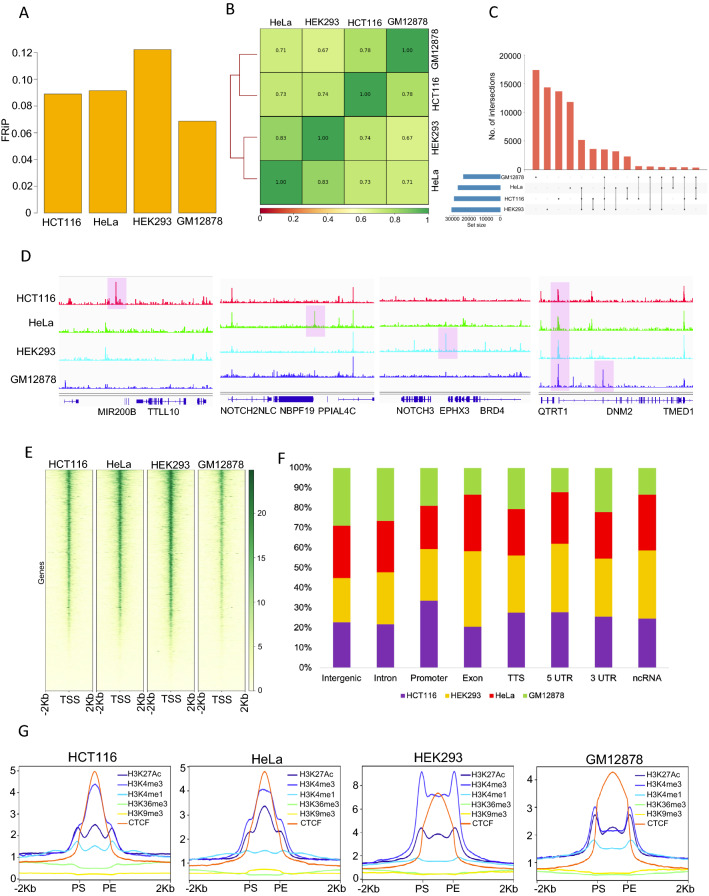
Comparison of accessible chromatin features using 25 cells from four different cell lines. **A** Comparison of FRiP scores of one-pot UniNicE-seq 25 cells in HCT116, HeLa, HEK293 and GM12878. **B** Correlation analysis of accessible chromatin regions in HCT116, HeLa, HEK293 and GM12878 using one-pot UniNicE-seq. **C** Upset plot showing common and unique accessible region peaks between HCT116, HeLa, HEK293 and GM12878 using one-pot UniNicE-seq. **D** Representative IGV screenshot of the normalized read density of the one-pot UniNicE-seq in HCT116, HeLa, HEK293 and GM12878 (scales remain same). **E** Heatmap showing signal intensity profile of TSS (that includes ± 2 Kb of flanking region) in HCT116, HeLa, HEK293 and GM12878 using one-pot UniNicE-seq. **F** Peak annotation of one-pot UniNicE-seq in HCT116, HeLa, HEK293 and GM12878. **G** Genome-wide signal intensity profile plot showing the enrichment of H3K27Ac, H3K4me3, H3K4me1, H3K36me3, H3K9me3 and CTCF in one-pot UniNicE-seq of HCT116, HeLa, HEK293 and GM12878. Peak-start and peak-end are noted as PS and PE

To further validate the distributions of accessible chromatin peaks in low cell numbers, we compared one-pot UniNicE-seq in 25 cells of HeLa, HCT116, HEK293 and GM12878 cell lines. The FRiP scores of the cell lines were between 0.07 and 0.12 (Fig. 4A). The Pearson’s correlation between reads from various cells ranged from 0.67 to 0.78, indicating similarity and differences between tissue-specific origin of cells (Fig. 4B). These similarity and differences were also observed in the called accessible peaks, particularly cell-type-specific peaks were more than the common peaks (Fig. 4C). IGV traces of different cell lines clearly demonstrated the unique and common accessible regions between different cell types (Fig. 4D). Furthermore, accessible chromatin peaks were reproducible using one-pot UniNicE-seq, DNase-seq, ATAC-seq and omni ATAC-seq in HCT116, HeLa, HEK293, and GM12878 demonstrating its applicability and showing cell line-specific accessible peaks (Additional file 1: Fig. S7). The accessible chromatin peaks were mostly concentrated at gene promoters, specifically transcription start sites, as expected (Fig. 4E, Additional file 1: Figs. S3C, S4C, S5C). The genic region of both introns and coding exons also displayed varying degrees of accessible chromatin independent of cell lines. Comparison of all accessible peaks representing genic features between cell lines for 25 cells essentially displayed a similar percentage of representation (Fig. 4F). In addition, a comparison between accessible chromatin heat map surrounding TSS/TTS in one-pot UniNicE-seq and RNA expression profile demonstrated the accessibility enrichment decreases concomitantly with the expression of transcripts (Additional file 1: Fig. S8).

Next, we also compared accessible chromatin peaks obtained by 25 cells with various active and inactive chromatin marks, and binder proteins. We extracted the distribution of tag densities for various ChIP-seq experiments (H3K4me1, H3K4me3, H3K27ac, H3K36me3, H3K9me3, and CTCF) in a ± 2-kb window around the identified accessible chromatin and generated heat maps. As expected, the transcriptionally active chromatin marks, H3K4me1, H3K4me3, H3K27ac and CTCF positively enriched at the accessible chromatin region and transcriptionally inactive chromatin marks H3K9me3 inversely correlated (Fig. 4G). Similarly, H3K36me3 mark that is pronounced in the gene body, appeared to be less accessible. The degree of correlation between accessible peaks obtained from 25 or 5 K cells remains indistinguishable confirming low cell numbers based accessible chromatin regions preserves both genic and epigenetic features.

### Comparison between formaldehyde fixed ATAC-see and low cell number one-pot universal NicE-seq

**Fig. 5:**
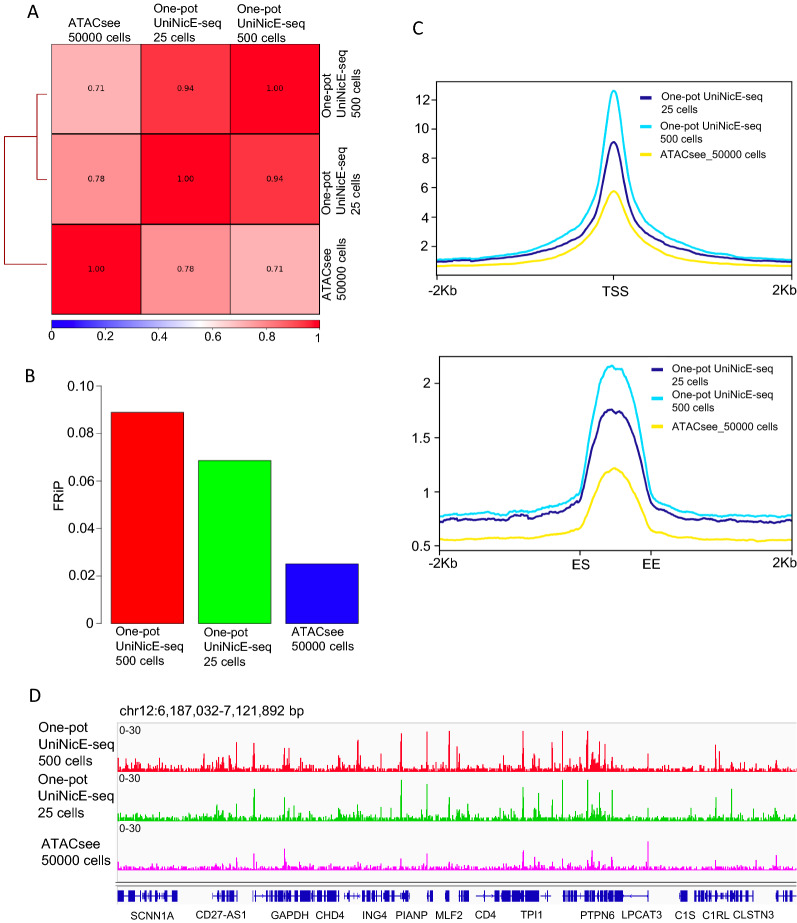
One-pot UniNicE-seq comparison with formaldehyde fixed ATAC-seq (ATAC-see) in GM12878 cells. **A** Genome-wide comparison of accessible chromatin between one-pot UniNicE-seq 25 and 500 cells with formaldehyde fixed ATAC-seq 50,000 cells (ATAC-see) using Pearson correlation. **B** Comparison of FRiP scores of one-pot UniNicE-seq 25 and 500 cells with fixed ATAC-seq 50,000 cells (ATAC-see). **C** Genome-wide metagene plot of TSS (top panel) and enhancer elements (bottom panel) with ± 2 Kb of flanking region of one-pot UniNicE-seq 25 cells, 500 cells and fixed ATAC-seq 50,000 cells. **D** Representative IGV screenshot of the normalized read density of the one-pot UniNicE-seq 25 cells, 500 cells and fixed ATAC-seq 50,000 cells

Accessible chromatin studies such as DNase-seq, FAIRE-seq, and NicE-seq use formaldehyde-fixed cells, compared to ATAC-seq that often uses unfixed cells. However, in a visualization and sequencing study, termed as ATAC-see, formaldehyde fixed GM12878 cells were used. We, therefore, performed one-pot UniNicE-seq on 25 and 500 fixed GM12878 cells and compared them with the published ATAC-see data sets. Pearson’s correlations between sequence reads for one-pot UniNicE-seq 25 or 500 cells and ATAC-see was 0.78 and 0.71, respectively, suggesting high similarity (Fig. 5A). Closer inspection of FRiP of ATAC-see data set demonstrated a 2.5–4.0 × lower compared to one-pot UniNicE-seq, suggesting Tn5 transposon-based accessible chromatin assay is relatively inefficient once the cells are fixed (Fig. 5B). Indeed, the read densities in both TSS ± 2.0 Kb and enhancer ± 2.0 Kb showed a lower enrichment for accessible chromatin in ATAC-see data sets, although 50,000 cells were used (Fig. 5C). The IGV signals for ATAC-see were lower compared to low cell number one-pot UniNicE-seq (Fig. 5D). These results suggest lower efficiency of Tn5 mediated tagmentation and accessible chromatin assay in ATAC-see compared to Nt.CviPII mediated one-pot UniNicE-seq.

### Comparison between 4% formaldehyde fixed one-pot UniNicE-seq with unfixed ATAC-seq and omni-ATAC-seq

We further compared 4% one-pot UniNicE-seq data sets of 25 and 500 cells with unfixed accessible chromatin methodologies, including ATAC-seq (50 K cells) and Omni-ATAC-seq (50 K cells) to investigate qualitative advantages of each method using HCT116 cells. All called accessible region peaks were compared using upset plot. There were about 13.4 K peaks common to all methods and large numbers of peaks remain method-specific (Additional file 1: Fig. S9A). The FRiP scores of ATAC-seq and omni-ATAC-seq were higher compared to one-pot UniNicE-seq (Additional file 1: Fig. S9B). This suggests that the unfixed cells yield efficiently more reads from accessible regions. However, metagene plots of TSS ± 2 Kb region and enhancer start and end sites ± 2 Kb regions yielded better signal for one-pot UniNicE-seq in 25 or 500 cells (Additional file 1: Fig. S9C). These observations led us to perform peak annotation to decipher the origin of all accessible peaks between different methods. Indeed, one-pot UniNicE-seq, ATAC-seq, and Omni-ATAC-seq had similar percentage representation in all the genomic features except promoters, where one-pot UniNicE-seq with smaller cell numbers displayed more read density (Additional file 1: Fig. S9D). Indeed, the IGV browser visual analysis of all these methods indicates no loss of accessible regions between methods (Additional file 1: Fig. S9E).

### Low cell number one-pot UniNicE-seq compared with aggregated scATAC-seq

**Fig. 6:**
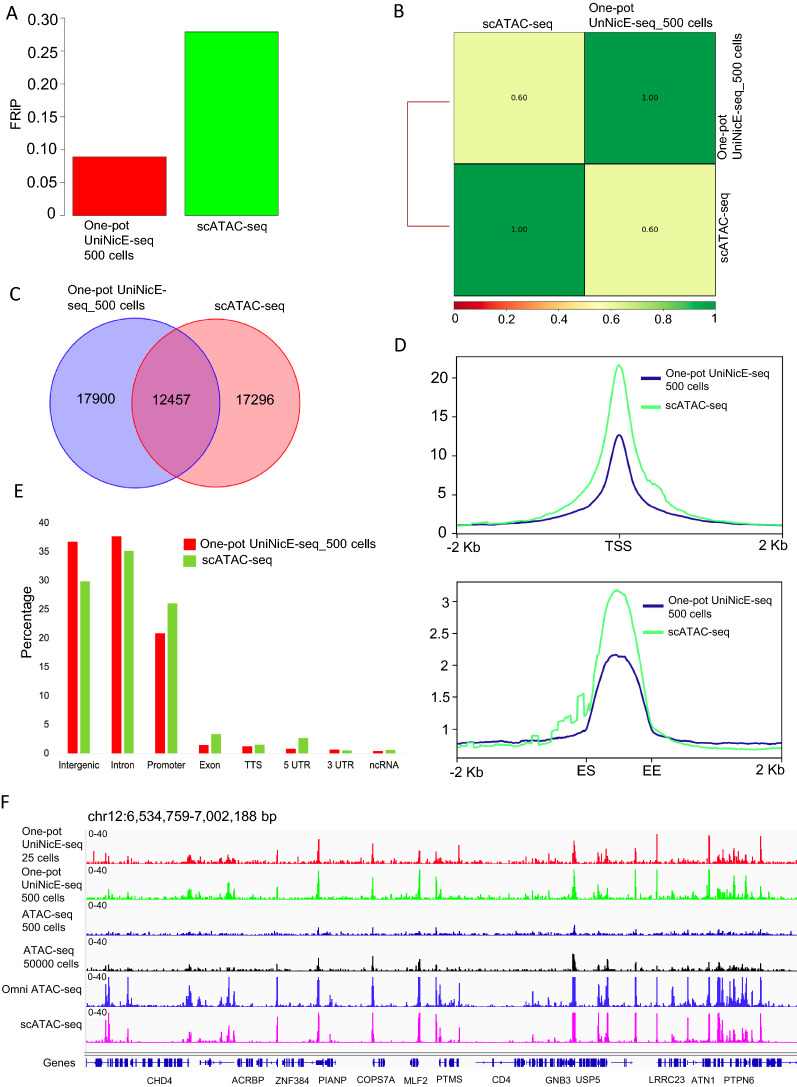
One-pot UniNicE-seq of 25 cells compared with scATAC-seq in GM12878 cells. **A** Comparison of FRiP scores of one-pot UniNicE-seq 500 cells and scATAC-seq. **B** Genome-wide comparison of accessible chromatin between one-pot UniNicE-seq 500 and scATAC-seq using Pearson correlation. **C** Venn diagram showing common and unique accessible region peaks between one-pot UniNicE-seq 500 cells and scATAC-seq. **D** Genome-wide metagene plot of TSS (top panel) and enhancer elements (bottom panel) with ± 2 Kb of flanking region of one-pot UniNicE-seq 500 and scATAC-seq. **E** Peak annotation of one-pot UniNicE-seq 500 and scATAC-seq. **F** Representative IGV genomic tracks of the normalized read density of the one-pot UniNicE-seq 500 cells, 25 cells, ATAC-seq, Omni ATAC-seq and scATAC-seq. All scATAC-seq data used for this analysis was derived from 384 cumulative cell reads

Next, we compared our lower cell number accessible chromatin data sets with the scATAC-seq data set of GM12878 cells. For example, a typical human scATAC-seq data set contains 100–1000 cells with 0.02–0.05 genome coverage per cell. However, the number of cis-regulatory elements in the genome exceeds sequence read coverage of single cell and thus are not represented in any mapped read. Furthermore, aggregated data from 384 individual GM12878 cells yielded an accessibility pattern to the pattern produced by population-based ATAC-seq. Therefore, we compared one-pot UniNicE-seq of 500 cells with 384 cells aggregated scATAC-seq. The FRiP score of 500 and scATAC-seq were 0.09 and 0.28, respectively, indicating higher numbers of reads in peak for aggregated scATAC-seq (Fig. 6A). The Pearson’s correlations between tag densities between one-pot UniNicE-seq and scATAC-seq was 0.60 demonstrating significant correlation (Fig. 6B). The Venn diagram of all accessible peaks represented about ~ 40% percentage of peaks being common for all data sets indicating cell-specific accessible regions are more prominent (Fig. 6C). Accessible chromatin tag density enrichment was better in promoter and enhancer in aggregated scATAC-seq, correlating with higher FRiP scores (Fig. 6D). However, the genomic features between methods showed that scATAC-seq is more efficient in promoter, exon and 5′ UTR capture compared to 500 cell one-pot UniNicE-seq. Similarly, 500 cell one-pot UniNicE-seq was more efficient in capturing accessible regions in intergenic and intron regions (Fig. 6E). The IGV tracks between methods were comparable suggesting these methods are both reproducible in low cell numbers (Fig. 6F).

## Discussion

Our one-pot universal NicE-seq with a low cell number can accurately determine the chromatin accessibility profile of mammalian cells as shown in this study. Due to low cell numbers, no DNA purification and sonication steps for DNA fragmentation, it allows all chemical processes to be carried out in one tube till on-bead library making. Furthermore, one-pot UniNicE-seq is shown to work well on either 1 or 4% formaldehyde fixing conditions. This fixative condition covers a wide array of samples covering from experimental biological laboratory samples with 1% formaldehyde fixation to clinical samples with 4% formaldehyde fixation for downstream processing. Although formaldehyde can damage DNA and has potential to adversely affect the DNA based analysis and interpretation of data, we found no evidence of it in our comparative analysis with non-fixed cells. Comparison between one-pot UniNicE-seq of 25 and 500 cells 4% formaldehyde fixed, with ATAC-seq or Omni ATAC-seq of 50,000 non-fixed cells resulted in similar patterns of accessible chromatin profiles in HCT116, HeLa, HEK293 and GM12878 cells (Additional file 1: Figs. S9E, S10A, B, Fig. 6F). Indeed, lower formaldehyde at 0.2% concentration led to DNA damage response pathways to be activated in the cell as demonstrated by poly ADP-ribosylation and H2AX histone accumulation. These cells also showed damaged DNA contributing to higher One-pot UniNicE-seq background. Therefore, 1–4% formaldehyde is an ideal concentration for cell fixation for accessible chromatin studies. Indeed, FAIRE-seq utilized 1% formaldehyde fixation for 1 to 7 min at 25 °C; and other methods such as ATAC-see and our original NicE-seq utilized 1% formaldehyde for 10 min. In routine chromatin immunoprecipitation experiments 1% formaldehyde is used. However, 4% formaldehyde was used in ADP-ribosylated DNA ChIP [25].

It is imperative that experimental manipulation of biological material such as mammalian cells ex vivo results in chromatin remodeling in response to change of environment. Therefore, snap fixation may be ideal in limiting any ex vivo biochemical processes that may affect chromatin accessibility and data interpretation. Transcription factors can bind with a range of affinity to the chromatin. Some weak binders may not remain bound to chromatin during sample preparation without fixation. For example, HMGB1 is loosely bound to chromatin and would require a simple micrococcal nuclease digestion to be released compared to BRD1 that would require 600 mM salt [26]. Comparison between transcription factor consensus binding site near the one-pot UniNicE-seq and ATAC-see, both formaldehyde fixed sample preparation, derived accessible chromatin-binding region displayed varying degrees of similarity and a contrast. A closer analysis of accessible chromatin using various sample preparation protocols used for ATAC-seq, scATAC-seq and ATAC-see also displayed varying degrees of similarity of transcription factor consensus binding site near accessible chromatin regions (Additional file 1: Fig. S11), confirming sample preparation may be a key determinant of reliable result [27]. In addition, fixed cells could be stored longer for future experiments eliminating the urgency of immediate sample processing.

## Conclusion

In summary, one-pot UniNicE-seq allows a rapid platform for accessible chromatin analysis without DNA purification and sonication and integrating accessible chromatin enzymatic enrichment, thus paving ways to rapid automation for multiple sample processing, library preparation, sequencing, and data analysis.

## Materials and methods

### Cell culture, DNA damage detection, confocal microscopy

HCT116, HeLa, HEK293 and GM12878 cells were grown according to ATCC’s recommendations. For the DNA damage study, HCT116 cells were cultured on micro cover glass (VWR #48366067) in a 6 well plate format and fixed for 10 min at RT with different percentages of formaldehyde added in 1X PBS. After formaldehyde removal, PBS with 0.125 M Glycine was added for 5 min at RT to quench the crosslinking reaction. Cells were then washed once with 1X PBS and incubated with 5% BSA (Sigma #A7906) for 1 h at RT in 0.1% Tween-PBS (T-PBS). Anti-phospho-histone H2A.X (Ser139) and anti-ADPribose antibodies (Cell Signaling Technology #9718S and Santa Cruz Biotechnology #sc-56198, respectively) were added overnight at 4 °C at the recommended manufacturer’s dilutions. After three times T-PBS washes for 5 min at RT, anti-rabbit IgG Alexa 488 or anti-mouse IgG Alexa 594 were added at 1/500 dilution in 5% BSA + T-PBS for 1 h at RT for phospho-histone H2AX (Ser139) or ADPribose detection, respectively. After three times T-PBS washes for 5 min at RT, the micro cover glass was mounted on microscope slides (VWR Vista Vision #16004-368) using Prolong Gold antifade reagent with DAPI (Thermo Fisher Scientific #P36935). Immunofluorescence (DNA damage detection and NicE-view labelling) was visualized using a LSM880 confocal microscope (Zeiss) and mean pixel intensity per channel was quantified on at least 80 individual cells for every formaldehyde fixation condition.

### Accessible chromatin labeling efficiency

Accessible chromatin labeling was done as described in Estève et al. [17]. Briefly, HCT116, HeLa, HEK293 and GM12878 cells were cross-linked using 0.2, 1 and 4% formaldehyde for 10 min at room temperature. Formaldehyde was quenched by 125 mM glycine. Cytoplasm was extracted by incubating the cross-linked cells in cytosolic buffer (15 mM Tris–HCl pH 7.5, 5 mM MgCl2, 0.5 mM DTT, 15 mM NaCl, 300 mM sucrose and 1% NP40) for 30 min on ice with occasional agitation. Slides were then washed twice with the cytosolic buffer. Fluorescent open chromatin DNA labeling was performed by incubating the nuclei in the presence of 2.5 U of Nt.CviPII (NEB R0626S), 50 U of DNA polymerase I (M0209S) and 30 μM of each dNTP including 6 μM of Fluorescein-12-dATP (Perkin Elmer, NEL465001EA) in 800 μl of 1 × NEBuffer 2 and carried out at 37 °C for 0 h, 0.5 h, 1 h and 2 h. 80 μl of 0.5 M EDTA and 2 μg of RNase A was added to the labeling reaction after each timepoint and incubated at 37 °C for 30 min to stop the labeling reaction and digest cellular RNA. After washing the slides once with PBS, a subsequent wash with PBS including 50 mM EDTA was performed at 55 °C for 15 min to remove fluorescent background followed by three washes with PBS for 5 min at RT. Slides were dried, mounted using Prolong Gold antifade reagent with DAPI (Invitrogen, P36935) and visualized using LSM 880 confocal microscope (Zeiss). Fluorescein-dATP and DAPI were detected using Argon 458, 488, 514 nm and diode 405 nm laser, respectively. Quantification of Fluorescein-dATP per nucleus was defined by mean pixel intensity per nucleus using the histogram and colocalization tools included in the Zen software (Zeiss).

### Effect of SDS in nicking enzyme activity

To evaluate the nicking enzyme activity in the presence of SDS, we performed a pilot experiment with plasmid DNA. pUC19 DNA (NEB, N3041S) was incubated in the presence of 2U of Nt.CviPII (NEB R0626S) and different SDS concentrations for 1 h at 37 °C in 50 µl 1X NEBuffer2. Then the DNA was loaded in to a 1% agarose gel and the gel was documented in Alpha imager HP. SDS was quenched with Triton X-100 (SIGMA, 93443). Different concentration of Triton was added along with SDS and the reaction was performed with Nt.CviPII as mentioned above.

### One-pot universal NicE-seq protocol

Accessible chromatin labeling was done as mentioned above with one modification, the incorporation of biotin-dCTP with other dNTPs during DNA labeling. Briefly, cells were grown to ∼75% confluency, harvested with trypsin, washed in 1× PBS. Cells were crosslinked with 4% formaldehyde for 10 min at room temperature in Eppendorf tubes. Formaldehyde quenching, cytoplasm extraction and accessible chromatin labeling was done as mentioned above. Then the cells were counted and 100,000 cells were used for open chromatin labeling. After 2 h of incubation at 37 °C, 0.5 M EDTA and 2 μg of RNase A was added to the labeling reaction and incubated at 37 °C for 30 min to stop the labeling reaction and digest cellular RNA. Then the labelled cells were serially diluted up to 25 cells. In this study, we used 5000, 1000, 500, 100 and 25 cells for the accessible chromatin analysis. Then the cells were spun at 1000 RPM for 5 min. The cell pellet was resuspended in 100 µl of 1X PBS with 0.1% SDS and 2 U of Thermolabile Proteinase K (TLPK, NEB P8111S) and incubated at 37 °C O/N. TLPK was inactivated by incubating at 55 °C for 10 min. Then 0.1% SDS was quenched with 1% Triton X-100 and the reaction was diluted tenfold using 1X NEBuffer2. The genomic DNA fragmentation was subsequently done using 10 U of Nt.CviPII at 37 °C for 4 h. After DNA fragmentation, Nt. CviPII was inactivated at 65 °C for 10 min. To capture biotin-labeled DNA, 30 µl of magnetic streptavidin beads (NEB, S1420S) were added and incubated at 4 °C for 2 h in the presence of 2 M NaCl. Then the library was prepared as mentioned in Chin et al. [16] with some modifications. Briefly, after the incubation of biotin-labeled DNA with magnetic streptavidin beads, the beads were washed twice with high salt buffer (10 mM Tris–HCl pH = 8, 2 M NaCl, 1 mM EDTA and 0.05% TritonX-100) and once with 1× TE buffer. Then the beads were resuspended in 50 µl of 0.1× low TE buffer. Then end-repair/dA tailing was done in a thermo cycler as mentioned in Chin et al. [16]. Then the beads were washed twice with 200 µl high salt buffer and once with 1× TE. Then the beads were resuspended in 60 µl of 0.1× low TE buffer. Then the adapter ligation, PCR amplification and library clean-up was done as mentioned in Chin et al. [16]. Sequencing was done on Illumina Nextseq 550 system.

### Data analysis

Read processing and data analysis was done as mentioned in Chin et al. [16] and Estève et al. [17]. Briefly, adapter and low-quality sequences from the raw FASTQ files were trimmed using Trim Galore (http://www.bioinformatics.babraham.ac.uk/projects/trim_galore/). Trimmed read pairs were mapped to the reference genome (human: hg38) using Bowtie2 [28]. PCR duplicates and mitochondrial reads were removed using samtools [29]. The aligned bam files of technical replicates were merged together using samtools. The merged aligned reads were downsized to same number of reads using sambamba [30]. MACS2 [31] was used to call the peaks from downsized aligned reads. The Fraction of reads in peaks (FRiP) score was calculated using the deepTools plotEnrichment function [32]. Peak overlap analysis, venn diagram and upset plot was generated using Bedtools [33], Intervene [34] and R [35]. Pearson Correlation analysis of NicE-seq open-chromatin signals were performed with the deeptools plotCorrelation function [32]. Here, the affinity-based correlation analysis was performed. In brief, the affinity-based method first determines the number of normalized reads that overlap with a set of all the merged peaks from individual samples and then calculates Pearson correlation based on the normalized read count matrix. Open chromatin peaks were annotated using HOMER annotatePeaks.pl [36]. HOMER annotates peaks as promoter (i.e., within 2 kb of known TSS), intergenic, intronic, exon, CpG islands, repetitive elements and other positional categories. TSS of human (hg38) genome were extracted from the NCBI RefGene gene table downloaded from the UCSC Table Browser. The cell line specific enhancers were downloaded from Enhancer Atlas 2.0 database [37]. ChIP-seq data sets of cell-specific CTCF binding and histone marks (H3K27ac, H3K36me3, H3K4me3, H3K4me1 and H3K9me3) were downloaded from human Encyclopedia of DNA Elements (ENCODE) projects and GEO. The TSS, enhancer, H3K27ac, H3K36me3, H3K4me3 H3K4me1, H3K9me3 and CTCF profiles was computed with the deeptools computeMatrix and plotheatmap and plotProfile functions. Transcription factor binding motifs enrichment near called open-chromatin peaks was searched using the HOMER tool findMotifsGenome.pl [36] with default parameters. The enrichment scores [− 10log(*P* value)] of individual TF binding motifs were calculated for one-pot UniNicE-seq, ATAC-seq, ATACsee and scATAC-seq and were summarized into a data matrix in R and a heatmap was then created to represent stage-specific enrichment of TF binding motifs near the open-chromatin peaks. ATAC-seq and Omni ATAC-seq data for HCT116, HEK293, HeLa and GM12878 cells and ATAC-see and scATAC-seq data for GM12878 were downloaded from GEO (Additional file 2: Table S1). Data processing for the external data sets was done as described above. Signal tracks were generated using 100 bp bins using deeptools bamCoverage with the following parameters – of bigwig – normalize Using RPKM. Genomic regions were visualized using Integrative genomic viewer (IGV) [38]. All data sets used here including their accession numbers are listed in Additional file 2: Table S1.

## Supplementary Information

**Additional file 1: Figure S1**. Different formaldehyde fixation in HCT116 cells. A) Comparison of FRiP scores of cells fixed with different formaldehyde concentration (0.2%, 1% and 4%). B) Pearson correlation of accessible chromatin between cells fixed with different formaldehyde concentration. C) Heatmap showing signal intensity profile of TSS (that includes ± 2 Kb of flanking region) in cells fixed with different formaldehyde concentration. D) Representative IGV genomic tracks of the normalized read density of cells fixed with different formaldehyde concentration. **Figure S2**. One-pot UniNicE-seq of HCT116 cells: A) Comparison of FRiP scores between replicates of one-pot UniNicE-seq 5000, 1000, 500, 100 and 25 cells. B) Pearson correlation of accessible chromatin peak read densities between the replicates of one-pot UniNicE-seq 5000 cells. C) Pearson correlation of accessible chromatin peak read densities between the replicates of one-pot UniNicE-seq 1000 cells. D) Pearson correlation of accessible chromatin peak read densities between the replicates of one-pot UniNicE-seq 500 cells. E) Pearson correlation of accessible chromatin peak read densities between the replicates of one-pot UniNicE-seq 100 cells. F) Pearson correlation of accessible chromatin peak read densities between the replicates of one-pot UniNicE-seq 25 cells. G) Heatmap showing signal intensity profile of TSS (that includes ± 2 Kb of flanking region) in the replicates of HCT116 5000, 1000, 500, 100 and 25 cells. **Figure S3.** One-pot UniNicE-seq of HeLa cells. A) Pearson correlation of accessible chromatin peak read densities between one-pot UniNicE-seq 5000, 1000, 500, 100 and 25 cells. B) Upset plot showing common and unique accessible region peaks between one-pot UniNicE-seq 5000, 1000, 500, 100 and 25 cells. C) Genome-wide metagene plot of TSS (top panel) and enhancer elements (bottom panel) with ± 2 Kb of flanking region of one-pot UniNicE-seq 5000, 1000, 500, 100 and 25 cells. Enhancer start (ES) and enhancer end (EE) site. D) Peak annotation of one-pot UniNicE-seq 5000, 1000, 500, 100 and 25 cells. E) Representative IGV genomic tracks of accessible chromatin of one-pot UniNicE-seq 5000, 1000, 500, 100 and 25 cells. **Figure S4.** One-pot UniNicE-seq of HEK293 cells. A) Pearson correlation of accessible chromatin peak read densities between one-pot UniNicE-seq 5000, 1000, 500, 100 and 25 cells. B) Upset plot showing common and unique accessible region peaks between one-pot UniNicE-seq 5000, 1000, 500, 100 and 25 cells. C) Genome-wide metagene plot of TSS (top panel) and enhancer elements (bottom panel) with ± 2 Kb of flanking region of one-pot UniNicE-seq 5000, 1000, 500, 100 and 25 cells. Enhancer start (ES) and enhancer end (EE) site. D) Peak annotation of one-pot UniNicE-seq 5000, 1000, 500, 100 and 25 cells. E) Representative IGV genomic tracks of accessible chromatin of one-pot UniNicE-seq 5000, 1000, 500, 100 and 25 cells. **Figure S5.** One-pot UniNicE-seq of GM12878 cells. A) Pearson correlation of accessible chromatin peak read densities between one-pot UniNicE-seq 5000, 1000, 500, 100 and 25 cells. B) Upset plot showing common and unique accessible region peaks between one-pot UniNicE-seq 5000, 1000, 500, 100 and 25 cells. C) Genome-wide metagene plot of TSS (top panel) and enhancer elements (bottom panel) with ± 2 Kb of flanking region of one-pot UniNicE-seq 5000, 1000, 500, 100 and 25 cells. Enhancer start (ES) and enhancer end (EE) site. D) Peak annotation of one-pot UniNicE-seq 5000, 1000, 500, 100 and 25 cells. E) Representative IGV genomic tracks of accessible chromatin of one-pot UniNicE-seq 5000, 1000, 500, 100 and 25 cells. **Figure S6.** Genomic distribution of the non-overlapping peaks in HCT116 cells using 5000, 1000, 500, 100 and 25 cells. **Figure S7.** Representative IGV genomic tracks of the normalized read density of the one-pot UniNicE-seq in HCT116, HeLa, HEK293 and GM12878 and comparison with ATAC-seq, Omni-ATAC-seq, DNase-seq. **Figure S8.** Heatmap showing signal intensity profile of hg38 genes (that includes ± 2 Kb of flanking region) in HCT116, HeLa, HEK293 and GM12878 using one-pot UniNicE-seq and RNA-seq. **Figure S9.** One-pot UniNicE-seq of 25 cells compared with ATAC-seq and Omni-ATAC-seq in HCT116 cells. A) Upset plot showing common and unique accessible region peaks between one-pot UniNicE-seq 500 cells, 25 cells and ATAC-seq and Omni-ATAC-seq. B) Comparison of FRiP scores of one-pot UniNicE-seq 500 cells, 25 cells and ATAC-seq and Omni ATAC-seq. C) Genome-wide metagene plot of TSS (top panel) and enhancer elements (bottom panel) with ± 2 Kb of flanking region of one-pot UniNicE-seq 500 cells, 25 cells and ATAC-seq and Omni-ATAC-seq. D) Peak annotation of one-pot UniNicE-seq 500 cells, 25 cells and ATAC-seq and Omni-ATAC-seq. E) Representative IGV genomic tracks of accessible chromatin using one-pot UniNicE-seq 500 cells, 25 cells and comparison with ATAC-seq and Omni-ATAC-seq. **Figure S10.** Representative IGV genomic tracks of accessible chromatin using one-pot UniNicE-seq and comparison with ATAC-seq and Omni-ATAC-seq in A) HeLa and B) HEK293 cells. **Figure S11.** Heatmap representing the enrichment of consensus TF-binding motifs identified in one-pot UniNicE-seq, ATAC-see, ATAC-seq, scATAC-seq derived accessible chromatin peaks. Both the TF binding motifs and the samples are organized by the unsupervised k-means clustering method. The *p* values of e − 6 were considered for the cluster analysis.

**Additional file 2: Table S1.** Data sets used in this study.

## Data Availability

One-pot UniNicE-seq data performed in this study are available in NCBI Gene Expression Omnibus (GEO) under the accession GSE175651. Further details can be obtained from corresponding author.

## Abbreviations

FFPE: Formalin-fixed paraffin-embedded
NicE-seq: Nicking enzyme assisted sequencing
UniNicE-seq: Universal nicking enzyme assisted sequencing
DNase-seq: DNase hypersensitive region sequencing
ATAC-seq: Assay for transposase‐accessible chromatin using sequencing
scATAC-seq: Single cell assay for transposase‐accessible chromatin using sequencing
FAIRE-seq: Formaldehyde-assisted isolation of regulatory elements
ChIP-seq: Chromatin immunoprecipitation sequencing
phospho-H2A.X: PH2A.X
5mdCTP: 5-Methyldeoxycytidine triphosphate
dNTP: Deoxynucleoside triphosphate
dATP: Deoxyadenosine triphosphate
dCTP: Deoxycytidine triphosphate
FRiP: Fraction of reads in peaks
DNA Pol I: DNA polymerase I
DHS: DNase hypersensitive sites
TSS: Transcription start sites
RPKM: Reads per kilobase of transcript, per million mapped reads
5′UTR: 5 Untranslated region
rRNA: Ribosomal RNA
PBS: Phosphate buffered saline
SDS: Sodium dodecyl sulphate
PCR: Polymerase chain reaction
CTCF: CCCTC-binding factor
ENCODE: Encyclopedia of DNA Elements
NCBI: National Center for Biotechnology Information
TLPK: Thermolabile proteinase K
IGV: Integrative genomic viewer
