# Supplementary material for: One-pot universal NicE-seq: all enzymatic downstream processing of 4% formaldehyde crosslinked cells for chromatin accessibility genomics
Source: Epigenetics Chromatin. 2021 Dec 11;14:53. doi: 10.1186/s13072-021-00427-2 (PMC8665596; doi:10.1186/s13072-021-00427-2)
Supplement: Supplementary file 1 — Additional file 1: Figure S1. Different formaldehyde fixation in HCT116 cells. A) Comparison of FRiP scores of cells fixed with different formaldehyde concentration (0.2%, 1% and 4%). B) Pearson correlation of accessible chromatin between cells fixed with different formaldehyde concentration. C) Heatmap showing signal intensity profile of TSS (that includes ± 2 Kb of flanking region) in cells fixed with different formaldehyde concentration. D) Representative IGV genomic tracks of the normalized read density of cells fixed with different formaldehyde concentration. Figure S2. One-pot UniNicE-seq of HCT116 cells: A) Comparison of FRiP scores between replicates of one-pot UniNicE-seq 5000, 1000, 500, 100 and 25 cells. B) Pearson correlation of accessible chromatin peak read densities between the replicates of one-pot UniNicE-seq 5000 cells. C) Pearson correlation of accessible chromatin peak read densities between the replicates of one-pot UniNicE-seq 1000 cells. D) Pearson correlation of accessible chromatin peak read densities between the replicates of one-pot UniNicE-seq 500 cells. E) Pearson correlation of accessible chromatin peak read densities between the replicates of one-pot UniNicE-seq 100 cells. F) Pearson correlation of accessible chromatin peak read densities between the replicates of one-pot UniNicE-seq 25 cells. G) Heatmap showing signal intensity profile of TSS (that includes ± 2 Kb of flanking region) in the replicates of HCT116 5000, 1000, 500, 100 and 25 cells. Figure S3. One-pot UniNicE-seq of HeLa cells. A) Pearson correlation of accessible chromatin peak read densities between one-pot UniNicE-seq 5000, 1000, 500, 100 and 25 cells. B) Upset plot showing common and unique accessible region peaks between one-pot UniNicE-seq 5000, 1000, 500, 100 and 25 cells. C) Genome-wide metagene plot of TSS (top panel) and enhancer elements (bottom panel) with ± 2 Kb of flanking region of one-pot UniNicE-seq 5000, 1000, 500, 100 and 25 cells. Enhancer start (ES) a [file 13072_2021_427_MOESM1_ESM.pptx]

## Slide 1
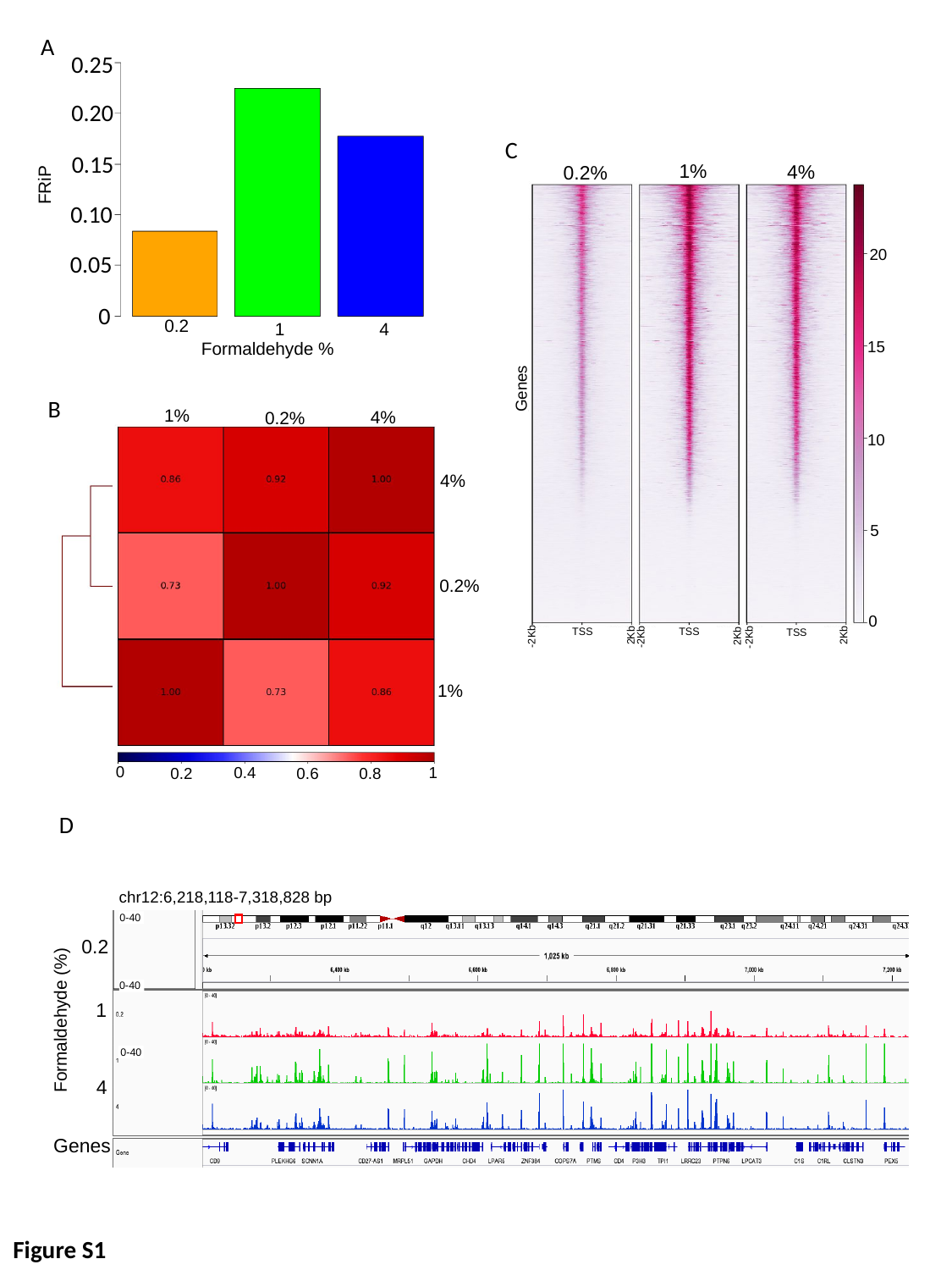

A
0.25
0.20
0.15
FRiP
0.10
0.05
0
0.2
1
4
C
1%
4%
0.2%
20
15
Genes
10
5
0
TSS
TSS
TSS
2Kb
2Kb
2Kb
-2Kb
-2Kb
-2Kb
Formaldehyde %
B
1%
4%
0.2%
4%
0.2%
1%
0
0.4
1
0.2
0.6
0.8
D
chr12:6,218,118-7,318,828 bp
0-40
0.2
1
Formaldehyde (%)
4
0-40
0-40
Genes
Figure S1

## Slide 2
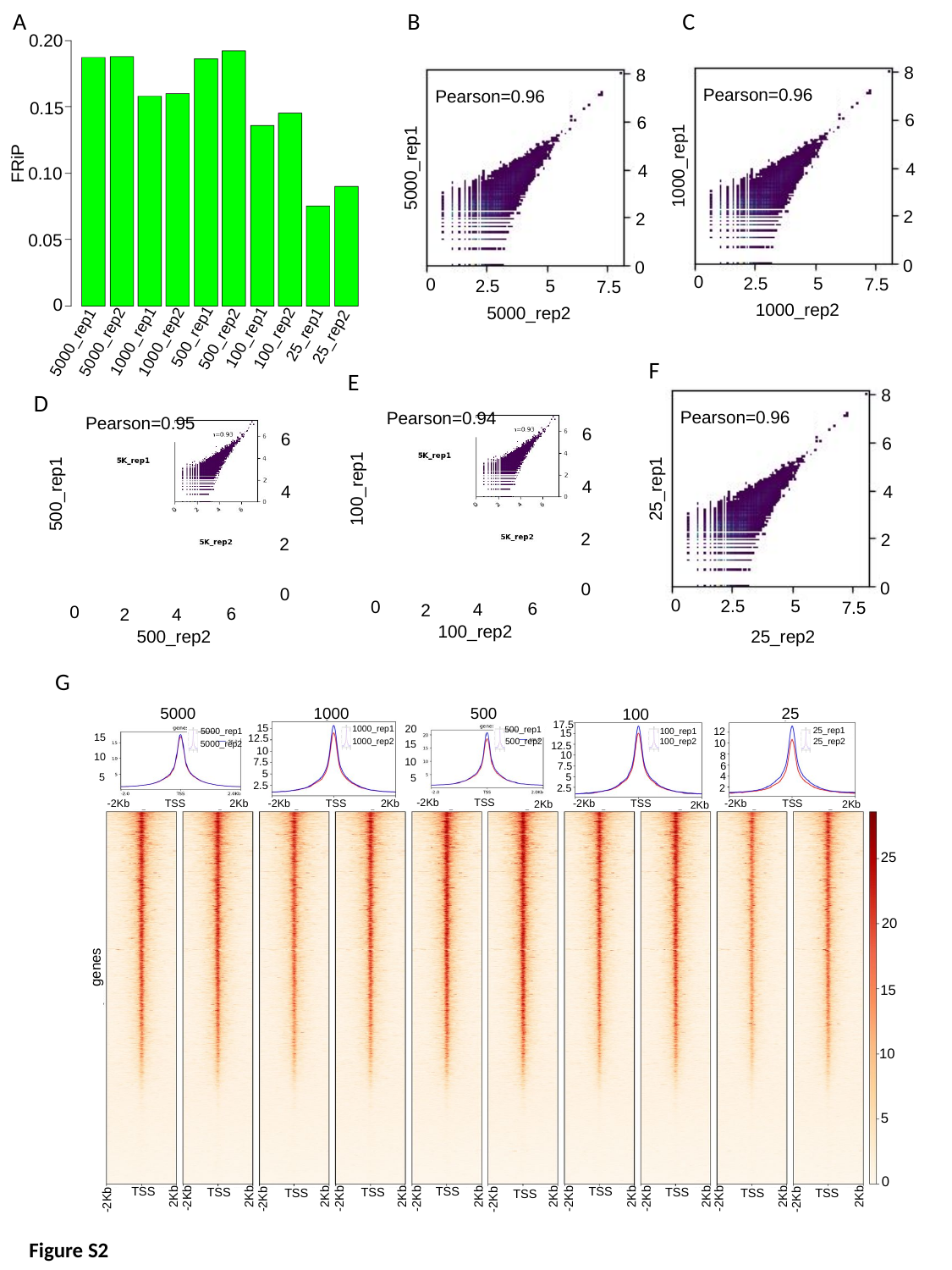

A
B
C
0.20
0.15
FRiP
0.10
0.05
0
25_rep1
500_rep1
25_rep2
100_rep1
100_rep2
500_rep2
1000_rep1
5000_rep2
1000_rep2
5000_rep1
8
Pearson=0.96
6
1000_rep1
4
2
0
0
5
2.5
7.5
1000_rep2
8
Pearson=0.96
6
5000_rep1
4
2
0
0
5
2.5
7.5
5000_rep2
F
E
8
Pearson=0.96
6
25_rep1
4
2
0
0
5
2.5
7.5
25_rep2
D
Pearson=0.94
6
4
100_rep1
2
0
0
6
4
2
100_rep2
Pearson=0.95
6
4
500_rep1
2
0
0
6
4
2
500_rep2
G
1000
500
5000
25
100
17.5
15
20
1000_rep1
500_rep1
25_rep1
12
100_rep1
5000_rep1
15
15
12.5
1000_rep2
100_rep2
500_rep2
25_rep2
10
15
5000_rep2
12.5
10
10
8
10
7.5
10
7.5
6
5
4
5
5
5
2.5
2
2.5
-2Kb
TSS
2Kb
-2Kb
TSS
-2Kb
TSS
2Kb
-2Kb
TSS
-2Kb
TSS
2Kb
2Kb
2Kb
25
20
genes
15
10
5
0
TSS
TSS
TSS
TSS
TSS
TSS
TSS
TSS
TSS
TSS
2Kb
2Kb
2Kb
2Kb
2Kb
2Kb
2Kb
2Kb
2Kb
2Kb
-2Kb
-2Kb
-2Kb
-2Kb
-2Kb
-2Kb
-2Kb
-2Kb
-2Kb
-2Kb
Figure S2

## Slide 3
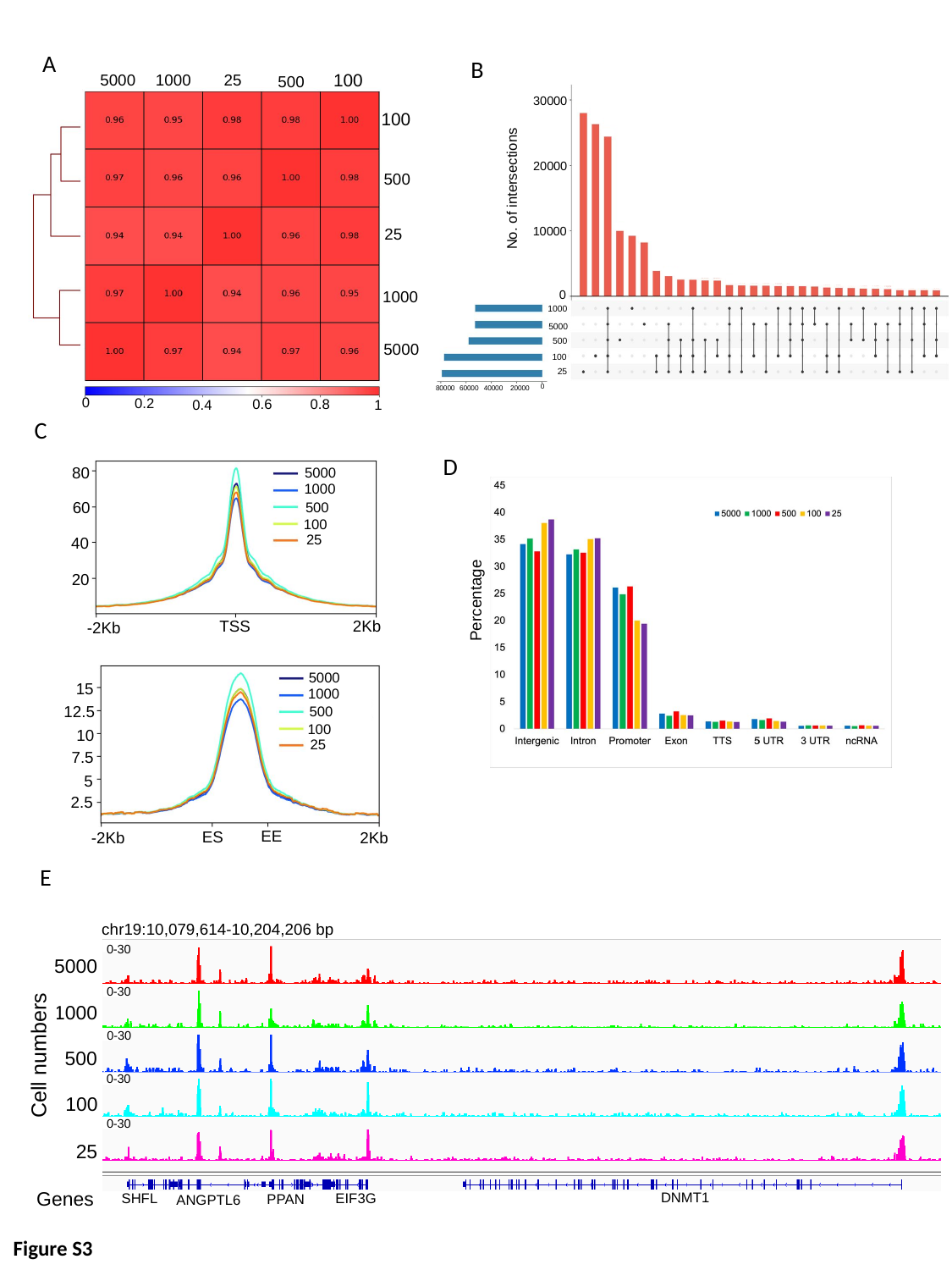

A
B
100
25
1000
5000
500
100
500
25
1000
5000
0
0.2
0.8
0.6
0.4
1
30000
20000
No. of intersections
10000
0
1000
5000
500
100
25
0
60000
40000
20000
80000
C
D
80
5000
1000
60
500
100
25
40
20
TSS
2Kb
-2Kb
Percentage
5000
15
1000
12.5
500
100
10
25
7.5
5
2.5
EE
ES
-2Kb
2Kb
E
chr19:10,079,614-10,204,206 bp
0-30
5000
1000
Cell numbers
500
100
25
Genes
0-30
0-30
0-30
0-30
DNMT1
SHFL
EIF3G
PPAN
ANGPTL6
Figure S3

## Slide 4
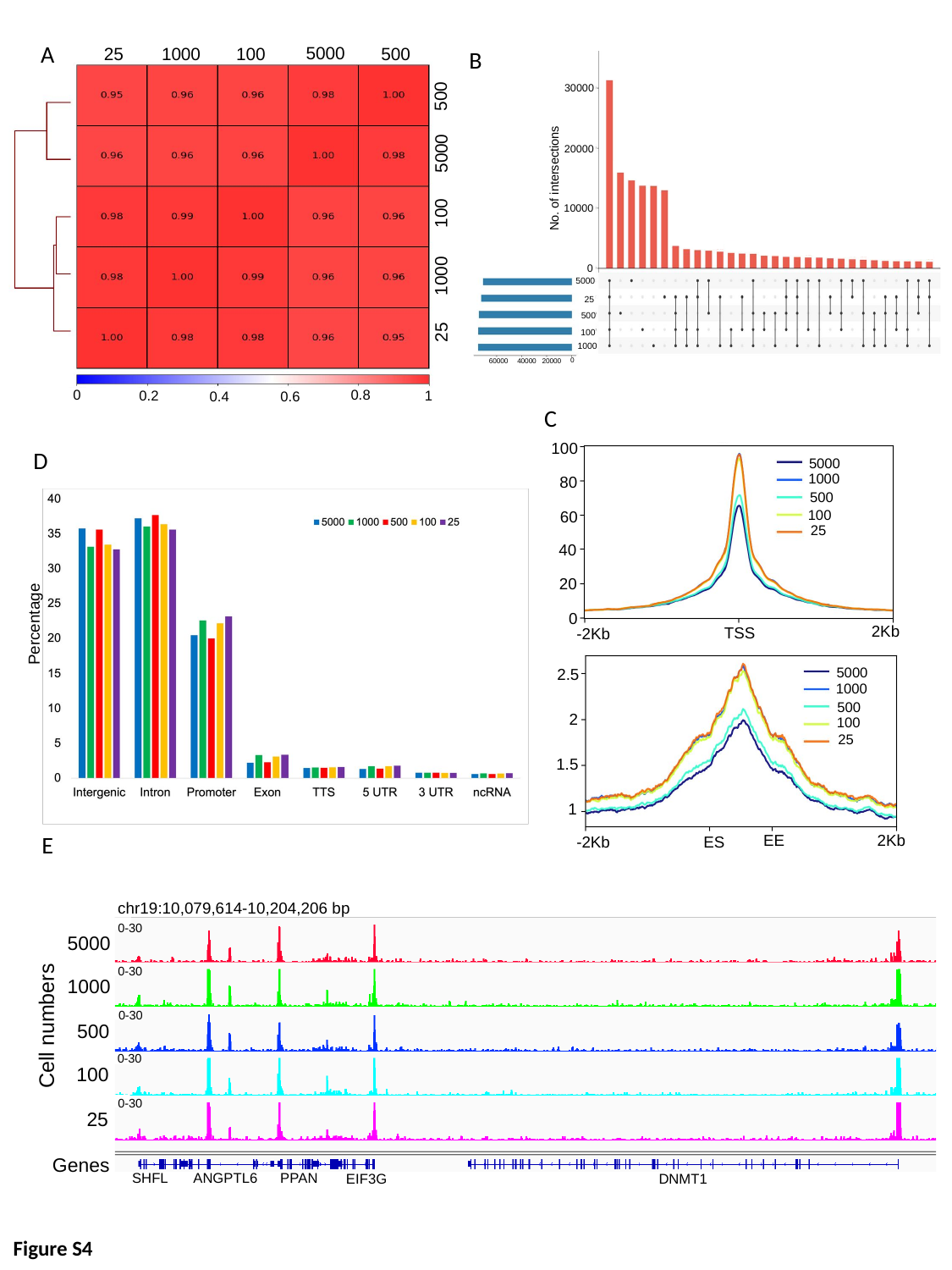

A
5000
25
500
100
1000
500
5000
100
1000
25
0.8
0
0.2
1
0.6
0.4
B
30000
20000
10000
0
5000
25
500
100
1000
0
40000
20000
60000
No. of intersections
C
100
5000
1000
80
500
100
60
25
40
20
0
2Kb
TSS
-2Kb
D
Percentage
5000
2.5
1000
500
2
100
25
1.5
1
EE
2Kb
ES
-2Kb
E
chr19:10,079,614-10,204,206 bp
0-30
5000
1000
Cell numbers
500
100
25
Genes
0-30
0-30
0-30
0-30
SHFL
PPAN
ANGPTL6
EIF3G
DNMT1
Figure S4

## Slide 5
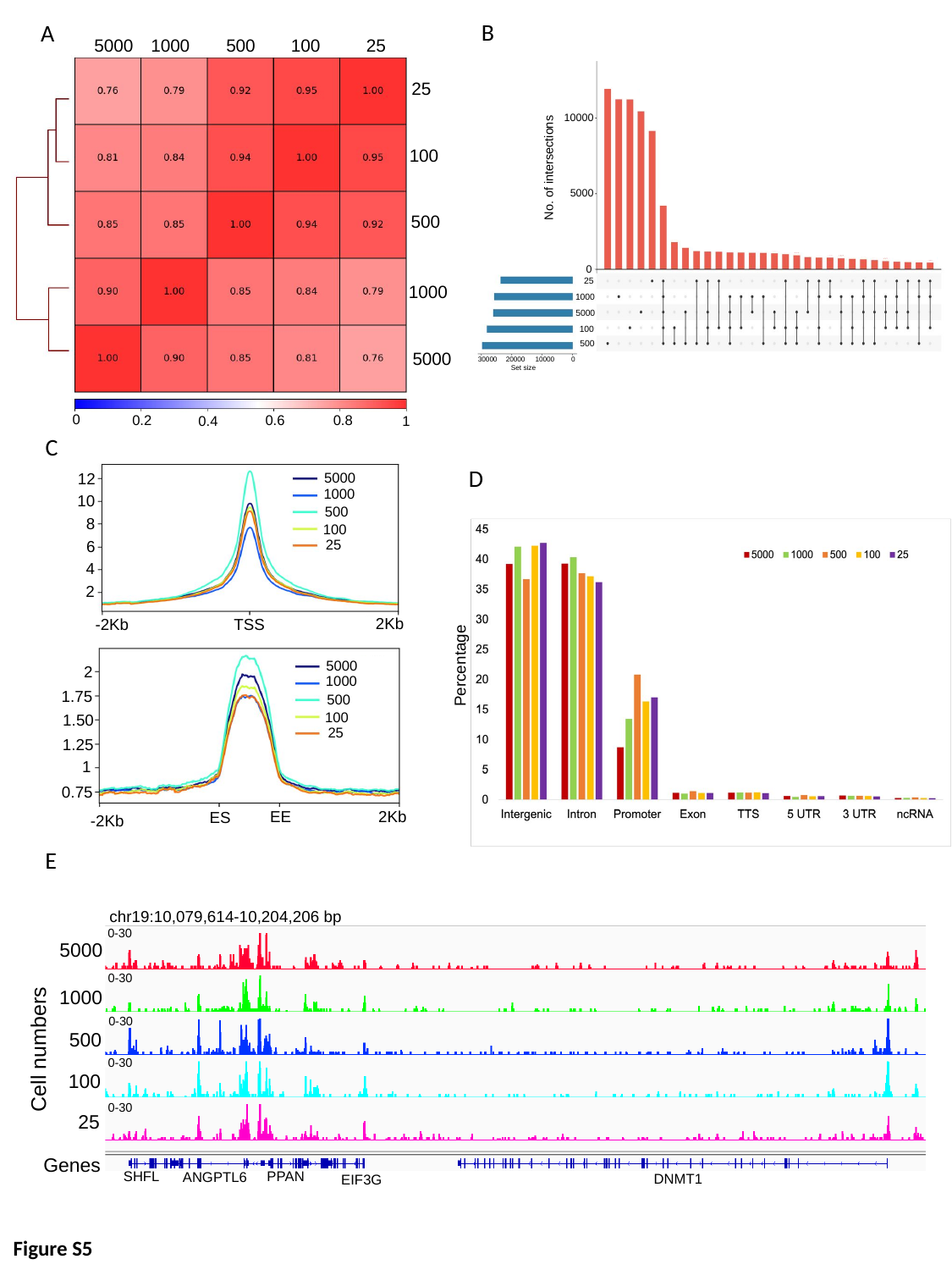

B
A
1000
100
5000
500
25
25
100
500
1000
5000
0
0.8
0.6
0.2
0.4
1
10000
No. of intersections
5000
0
25
1000
5000
100
500
20000
10000
0
Set size
30000
C
D
5000
12
1000
10
500
8
100
25
6
4
2
2Kb
TSS
-2Kb
5000
2
1000
1.75
500
100
1.50
25
1.25
1
0.75
EE
2Kb
ES
-2Kb
Percentage
E
chr19:10,079,614-10,204,206 bp
0-30
5000
1000
500
Cell numbers
100
25
Genes
0-30
0-30
0-30
0-30
SHFL
PPAN
ANGPTL6
DNMT1
EIF3G
Figure S5

## Slide 6
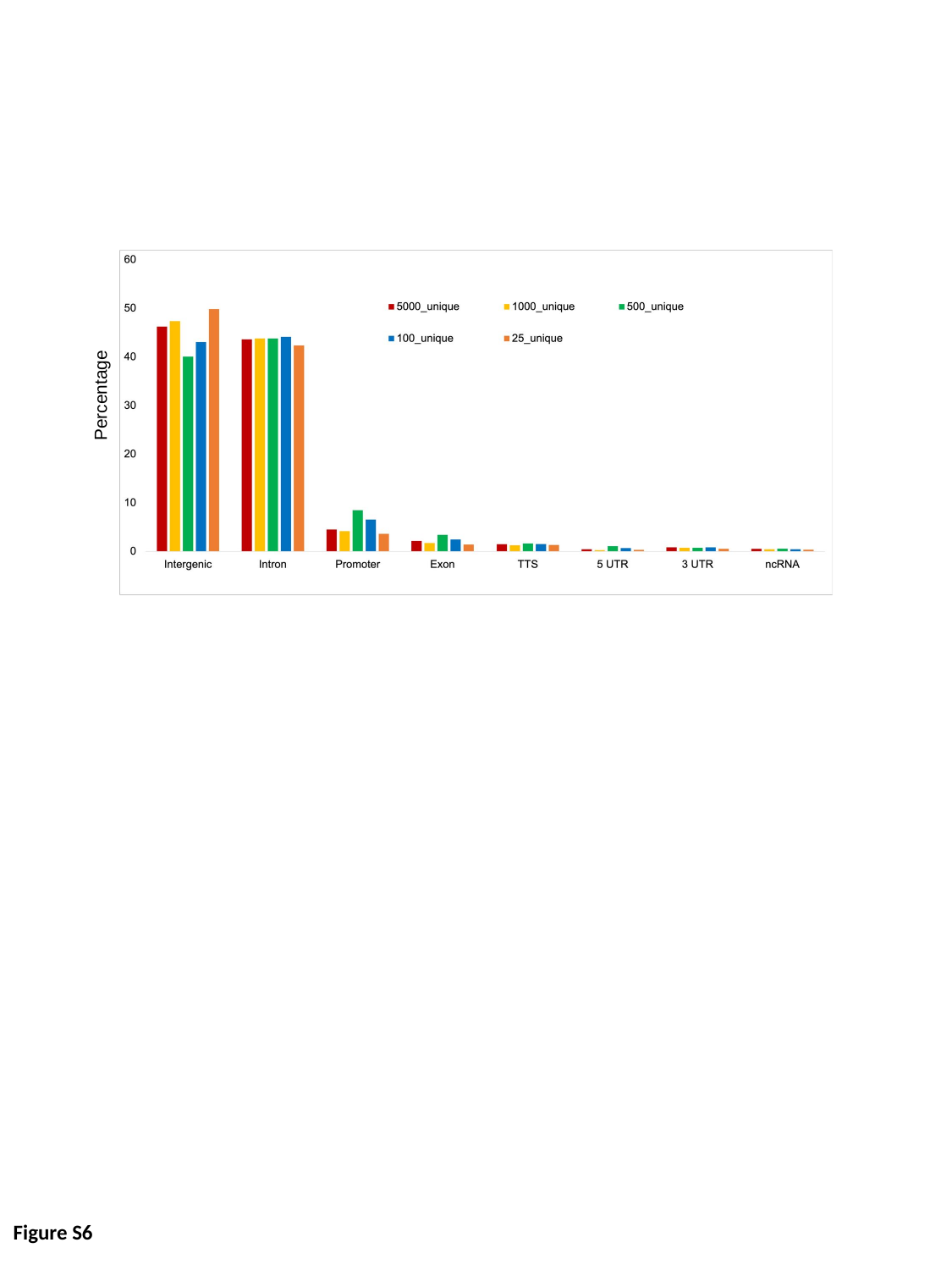

Percentage
Figure S6

## Slide 7
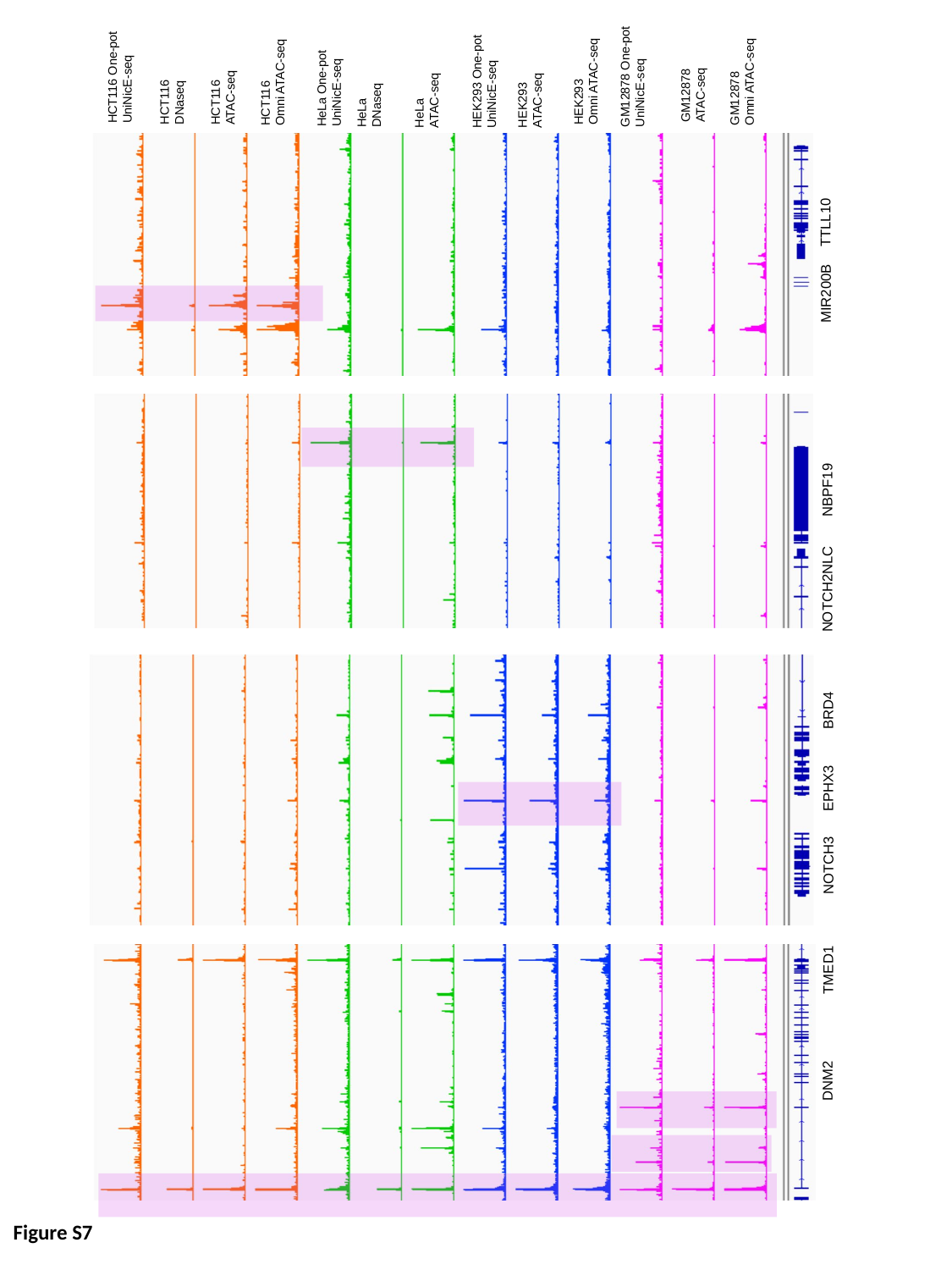

GM12878 One-pot
UniNicE-seq
GM12878
ATAC-seq
HCT116
ATAC-seq
HeLa
ATAC-seq
HEK293
ATAC-seq
HCT116
DNaseq
HeLa
DNaseq
TTLL10
MIR200B
NBPF19
NOTCH2NLC
BRD4
EPHX3
NOTCH3
TMED1
DNM2
HCT116 One-pot
UniNicE-seq
HEK293 One-pot
UniNicE-seq
HEK293
Omni ATAC-seq
GM12878
Omni ATAC-seq
HCT116
Omni ATAC-seq
HeLa One-pot
UniNicE-seq
Figure S7

## Slide 8
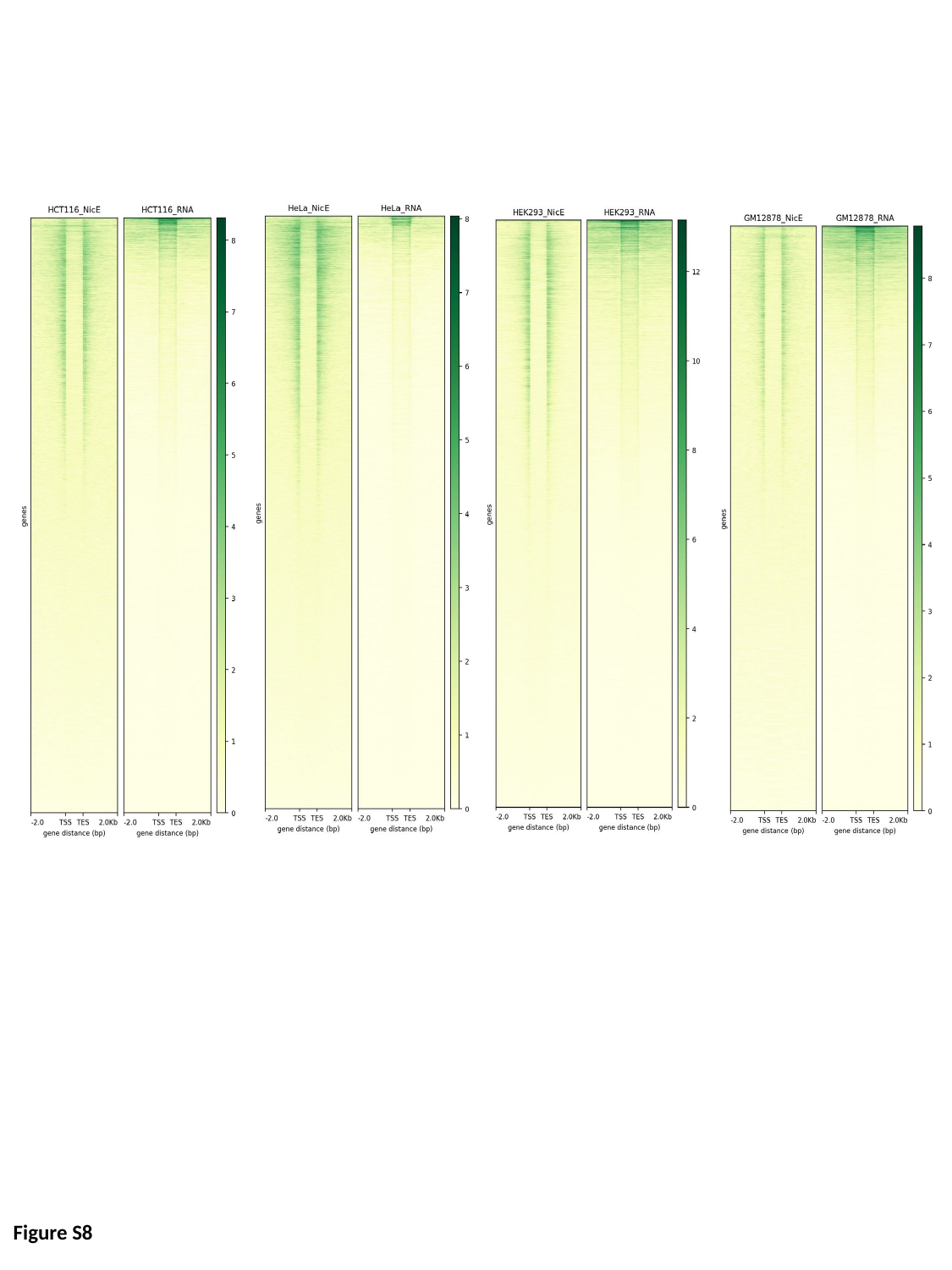

Figure S8

## Slide 9
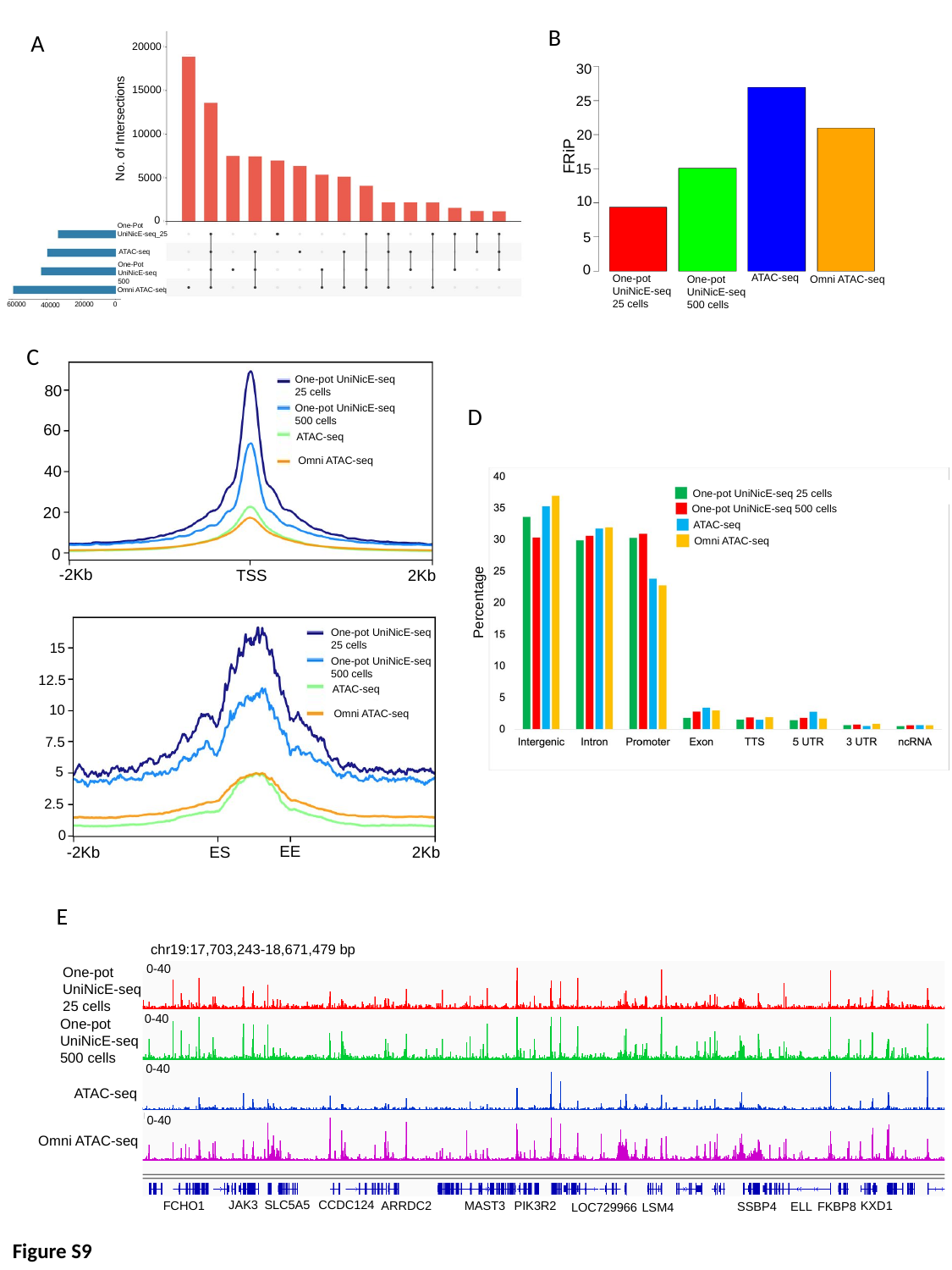

B
A
20000
15000
No. of Intersections
10000
5000
0
One-Pot
UniNicE-seq_25
ATAC-seq
One-Pot
UniNicE-seq
500
Omni ATAC-seq
0
60000
20000
40000
30
25
20
FRiP
15
10
5
0
ATAC-seq
Omni ATAC-seq
One-pot
UniNicE-seq
25 cells
One-pot
UniNicE-seq
500 cells
C
One-pot UniNicE-seq
25 cells
80
One-pot UniNicE-seq
500 cells
60
ATAC-seq
Omni ATAC-seq
40
20
0
-2Kb
TSS
2Kb
D
One-pot UniNicE-seq 25 cells
One-pot UniNicE-seq 500 cells
ATAC-seq
Omni ATAC-seq
Percentage
One-pot UniNicE-seq
25 cells
15
One-pot UniNicE-seq
500 cells
12.5
ATAC-seq
10
Omni ATAC-seq
7.5
5
2.5
0
EE
2Kb
ES
-2Kb
E
chr19:17,703,243-18,671,479 bp
0-40
One-pot
UniNicE-seq
25 cells
ATAC-seq
Omni ATAC-seq
JAK3
SLC5A5
CCDC124
KXD1
FCHO1
ARRDC2
MAST3
PIK3R2
SSBP4
ELL
FKBP8
LOC729966
LSM4
0-40
One-pot
UniNicE-seq
500 cells
0-40
0-40
Figure S9

## Slide 10
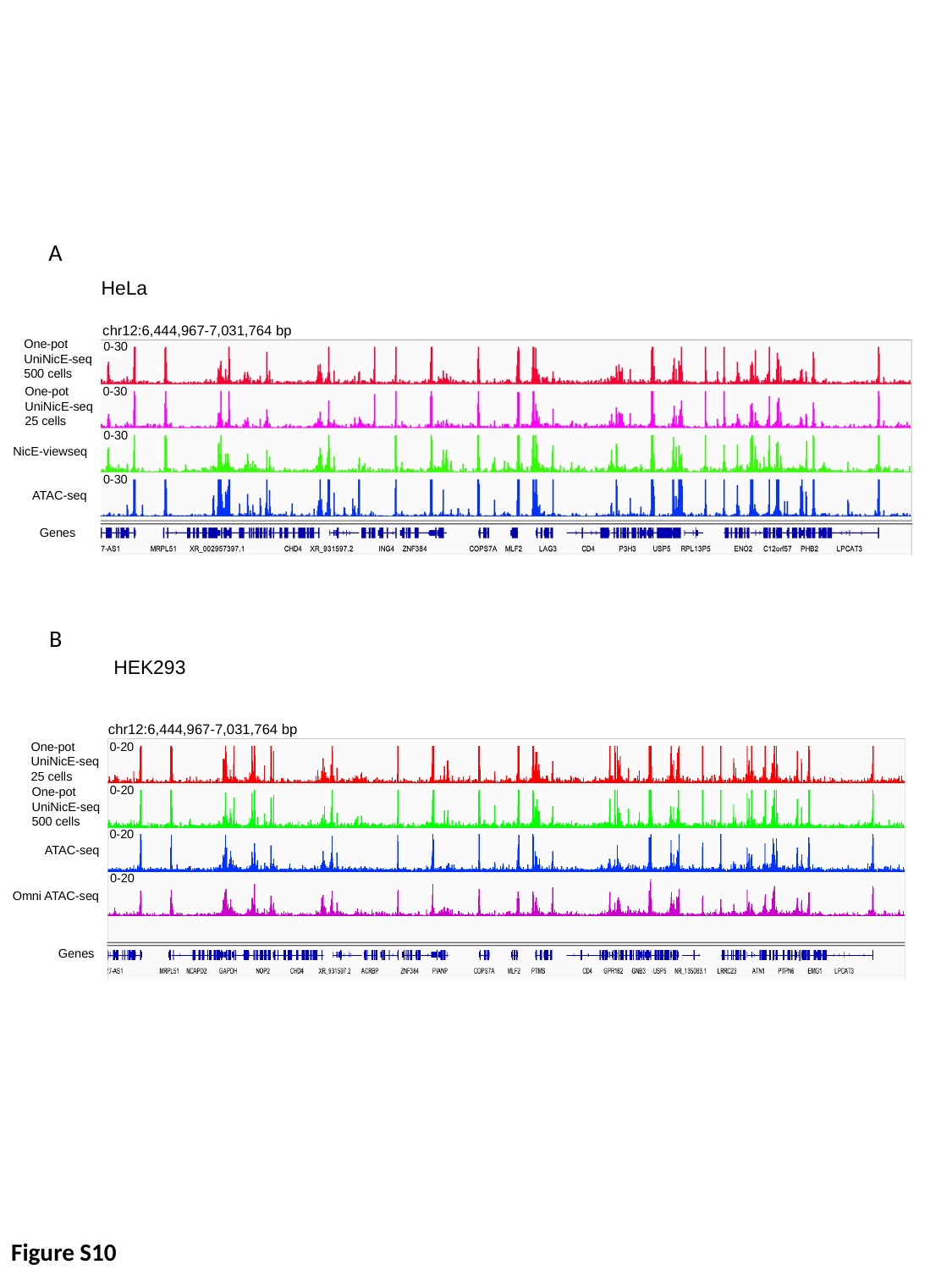

A
HeLa
chr12:6,444,967-7,031,764 bp
One-pot
UniNicE-seq
500 cells
One-pot
UniNicE-seq
25 cells
NicE-viewseq
ATAC-seq
Genes
0-30
0-30
0-30
0-30
B
HEK293
chr12:6,444,967-7,031,764 bp
0-20
One-pot
UniNicE-seq
25 cells
One-pot
UniNicE-seq
500 cells
ATAC-seq
Omni ATAC-seq
Genes
0-20
0-20
0-20
Figure S10

## Slide 11
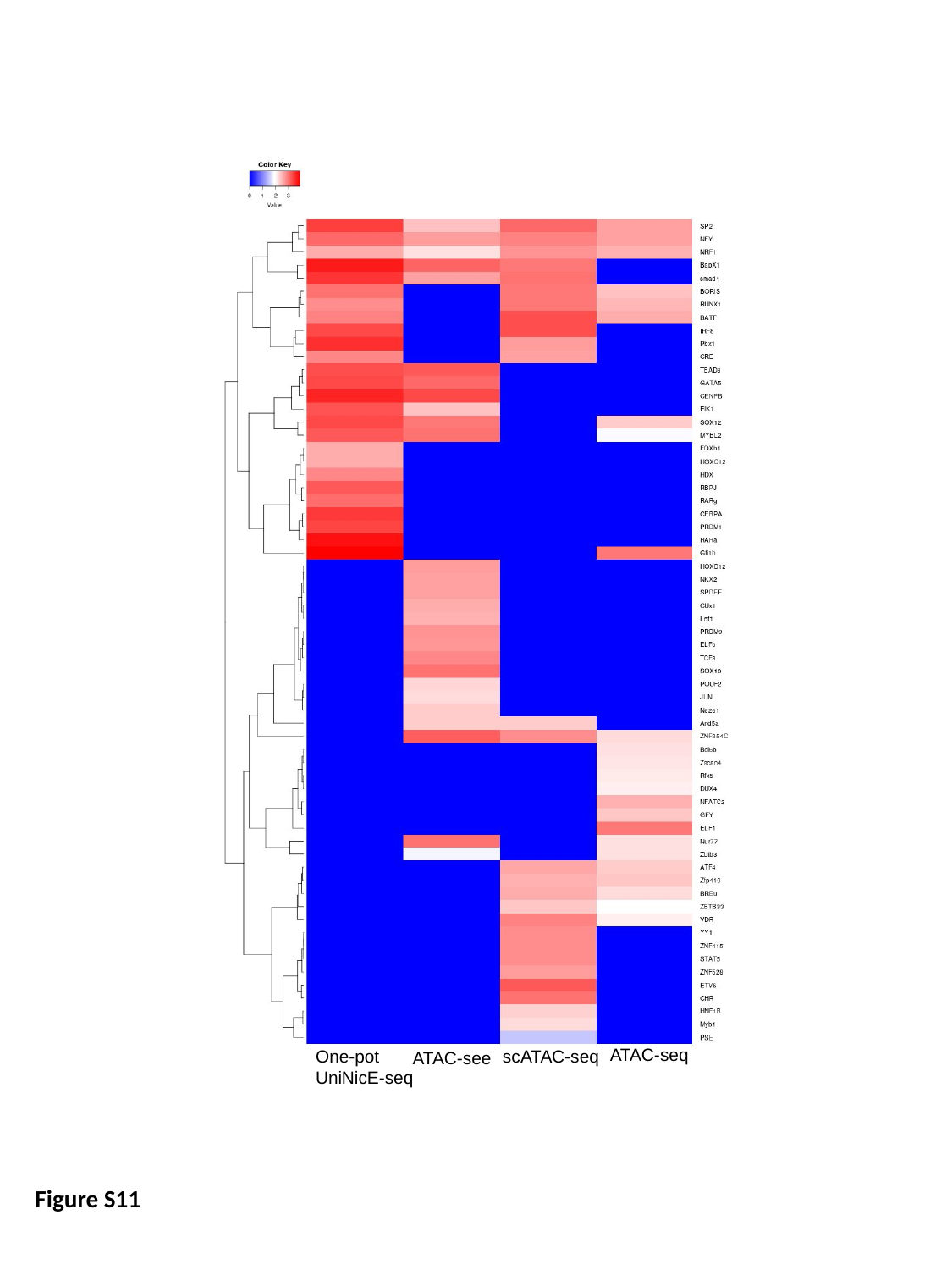

ATAC-seq
scATAC-seq
One-pot
UniNicE-seq
ATAC-see
Figure S11
