# Supplementary material for: One-pot universal NicE-seq: all enzymatic downstream processing of 4% formaldehyde crosslinked cells for chromatin accessibility genomics
Source: Epigenetics Chromatin. 2021 Dec 11;14:53. doi: 10.1186/s13072-021-00427-2 (PMC8665596; doi:10.1186/s13072-021-00427-2)
Supplement: Supplementary file 2 — Additional file 2: Table S1. Data sets used in this study. [file 13072_2021_427_MOESM2_ESM.docx]

**Table S1: Data sets used in this study**

| Cell line/ Tissue/ Patient samples | Condition/Antibody and Replicate number | Experiment | Single End (SE) or Paired End (PE) | Genome | Source | Accession |
| --- | --- | --- | --- | --- | --- | --- |
| HCT116 | 0.2% Formaldehyde_fixed_1 | One-pot UniNicE-seq | PE | hg38 | This study | GSE175651 |
| HCT116 | 0.2% Formaldehyde_fixed_2 | One-pot UniNicE-seq | PE | hg38 | This study | GSE175651 |
| HCT116 | 1% Formaldehyde_fixed_1 | One-pot UniNicE-seq | PE | hg38 | This study | GSE175651 |
| HCT116 | 1% Formaldehyde_fixed_2 | One-pot UniNicE-seq | PE | hg38 | This study | GSE175651 |
| HCT116 | 4% Formaldehyde_fixed_1 | One-pot UniNicE-seq | PE | hg38 | This study | GSE175651 |
| HCT116 | 4% Formaldehyde_fixed_2 | One-pot UniNicE-seq | PE | hg38 | This study | GSE175651 |
| HCT116 | 5000_cells_1 | One-pot UniNicE-seq | PE | hg38 | This study | GSE175651 |
| HCT116 | 5000_cells_2 | One-pot UniNicE-seq | PE | hg38 | This study | GSE175651 |
| HCT116 | 1000_cells_1 | One-pot UniNicE-seq | PE | hg38 | This study | GSE175651 |
| HCT116 | 1000_cells_2 | One-pot UniNicE-seq | PE | hg38 | This study | GSE175651 |
| HCT116 | 500_cells_1 | One-pot UniNicE-seq | PE | hg38 | This study | GSE175651 |
| HCT116 | 500_cells_2 | One-pot UniNicE-seq | PE | hg38 | This study | GSE175651 |
| HCT116 | 100_cells_1 | One-pot UniNicE-seq | PE | hg38 | This study | GSE175651 |
| HCT116 | 100_cells_2 | One-pot UniNicE-seq | PE | hg38 | This study | GSE175651 |
| HCT116 | 25_cells_1 | One-pot UniNicE-seq | PE | hg38 | This study | GSE175651 |
| HCT116 | 25_cells_2 | One-pot UniNicE-seq | PE | hg38 | This study | GSE175651 |
| HEK293 | 5000_cells_1 | One-pot UniNicE-seq | PE | hg38 | This study | GSE175651 |
| HEK293 | 5000_cells_2 | One-pot UniNicE-seq | PE | hg38 | This study | GSE175651 |
| HEK293 | 1000_cells_1 | One-pot UniNicE-seq | PE | hg38 | This study | GSE175651 |
| HEK293 | 1000_cells_2 | One-pot UniNicE-seq | PE | hg38 | This study | GSE175651 |
| HEK293 | 500_cells_1 | One-pot UniNicE-seq | PE | hg38 | This study | GSE175651 |
| HEK293 | 500_cells_2 | One-pot UniNicE-seq | PE | hg38 | This study | GSE175651 |
| HEK293 | 100_cells_1 | One-pot UniNicE-seq | PE | hg38 | This study | GSE175651 |
| HEK293 | 100_cells_2 | One-pot UniNicE-seq | PE | hg38 | This study | GSE175651 |
| HEK293 | 25_cells_1 | One-pot UniNicE-seq | PE | hg38 | This study | GSE175651 |
| HEK293 | 25_cells_2 | One-pot UniNicE-seq | PE | hg38 | This study | GSE175651 |
| HeLa | 5000_cells_1 | One-pot UniNicE-seq | PE | hg38 | This study | GSE175651 |
| HeLa | 1000_cells_1 | One-pot UniNicE-seq | PE | hg38 | This study | GSE175651 |
| HeLa | 1000_cells_2 | One-pot UniNicE-seq | PE | hg38 | This study | GSE175651 |
| HeLa | 500_cells_1 | One-pot UniNicE-seq | PE | hg38 | This study | GSE175651 |
| HeLa | 500_cells_2 | One-pot UniNicE-seq | PE | hg38 | This study | GSE175651 |
| HeLa | 100_cells_1 | One-pot UniNicE-seq | PE | hg38 | This study | GSE175651 |
| HeLa | 100_cells_2 | One-pot UniNicE-seq | PE | hg38 | This study | GSE175651 |
| HeLa | 25_cells_1 | One-pot UniNicE-seq | PE | hg38 | This study | GSE175651 |
| HeLa | 25_cells_2 | One-pot UniNicE-seq | PE | hg38 | This study | GSE175651 |
| GM12878 | 5000_cells_1 | One-pot UniNicE-seq | PE | hg38 | This study | GSE175651 |
| GM12878 | 5000_cells_2 | One-pot UniNicE-seq | PE | hg38 | This study | GSE175651 |
| GM12878 | 1000_cells_1 | One-pot UniNicE-seq | PE | hg38 | This study | GSE175651 |
| GM12878 | 1000_cells_2 | One-pot UniNicE-seq | PE | hg38 | This study | GSE175651 |
| GM12878 | 500_cells_1 | One-pot UniNicE-seq | PE | hg38 | This study | GSE175651 |
| GM12878 | 500_cells_2 | One-pot UniNicE-seq | PE | hg38 | This study | GSE175651 |
| GM12878 | 100_cells_1 | One-pot UniNicE-seq | PE | hg38 | This study | GSE175651 |
| GM12878 | 100_cells_2 | One-pot UniNicE-seq | PE | hg38 | This study | GSE175651 |
| GM12878 | 25_cells_1 | One-pot UniNicE-seq | PE | hg38 | This study | GSE175651 |
| GM12878 | 25_cells_2 | One-pot UniNicE-seq | PE | hg38 | This study | GSE175651 |
| HCT116 | CTCF | ChIP-seq | SE | hg38 | Gertz et al., 2013 [1] | ENCSR000BSE |
| HCT116 | H3K27Ac | ChIP-seq | SE/PE | hg38 | ENCODE project consortium, 2012 [2] | ENCSR661KMA |
| HCT116 | H3K4me3 | ChIP-seq | SE/PE | hg38 | ENCODE project consortium, 2012 [2] | ENCSR333OPW |
| HCT116 | H3K36me3 | ChIP-seq | SE/PE | hg38 | ENCODE project consortium, 2012 [2] | ENCSR091QXP |
| HCT116 | H3K4me1 | ChIP-seq | SE/PE | hg38 | ENCODE project consortium, 2012 [2] | ENCSR161MXP |
| HCT116 | H3K9me3 | ChIP-seq | SE/PE | hg38 | ENCODE project consortium, 2012 [2] | ENCSR179BUC |
| HEK293 | CTCF | ChIP-seq | PE | hg38 | ENCODE project consortium, 2012 [2] | ENCSR617IFZ |
| HEK293 | H3K27Ac | ChIP-seq | SE | hg38 | Frietze et al., 2012 [3] | ENCSR000FCH |
| HEK293 | H3K4me3 | ChIP-seq | SE | hg38 | ENCODE project consortium, 2012 [2] | ENCSR000DTU |
| HEK293 | H3K36me3 | ChIP-seq | SE | hg38 | ENCODE project consortium, 2012 [2] | ENCSR000FCK |
| HEK293 | H3K4me1 | ChIP-seq | SE | hg38 | Frietze et al., 2012 [3] | ENCSR000FCG |
| HEK293 | H3K9me3 | ChIP-seq | SE | hg38 | ENCODE project consortium, 2012 [2] | ENCSR000FCJ |
| HeLa | CTCF | ChIP-seq | SE | hg38 | ENCODE project consortium, 2012 [2] | ENCSR000AOA |
| HeLa | H3K27Ac | ChIP-seq | SE | hg38 | ENCODE project consortium, 2012 [2] | ENCSR000AOC |
| HeLa | H3K4me3 | ChIP-seq | SE/PE | hg38 | ENCODE project consortium, 2012 [2] | ENCSR340WQU |
| HeLa | H3K36me3 | ChIP-seq | SE | hg38 | ENCODE project consortium, 2012 [2] | ENCSR000AOD |
| HeLa | H3K4me1 | ChIP-seq | SE | hg38 | ENCODE project consortium, 2012 [2] | ENCSR000APW |
| HeLa | H3K9me3 | ChIP-seq | SE | hg38 | ENCODE project consortium, 2012 [2] | ENCSR000AQO |
| GM12878 | CTCF | ChIP-seq | SE | hg38 | ENCODE project consortium, 2012 [2] | ENCSR000DZN |
| GM12878 | H3K27Ac | ChIP-seq | SE | hg38 | ENCODE project consortium, 2012 [2] | ENCSR000AKC |
| GM12878 | H3K4me3 | ChIP-seq | SE | hg38 | ENCODE project consortium, 2012 [2] | ENCSR057BWO |
| GM12878 | H3K36me3 | ChIP-seq | SE | hg38 | ENCODE project consortium, 2012 [2] | ENCSR000AKE |
| GM12878 | H3K4me1 | ChIP-seq | SE | hg38 | ENCODE project consortium, 2012 [2] | ENCSR000AKF |
| GM12878 | H3K9me3 | ChIP-seq | SE | hg38 | ENCODE project consortium, 2012 [2] | ENCSR000AOX |
| HeLa | Control | ATAC-seq | PE | hg38 | Cho et al.,2018 [4] | GSE106145 |
| HeLa | Control | NicEview-seq | PE | hg38 | Pierre et al., 2020 (17)* | GSE139253 |
| HeLa | Control | DNase-seq | PE | hg38 | ENCODE project consortium, 2012 [2] | ENCSR959ZXU |
| HeLa | Control | RNA-seq | PE | hg38 |  | GSE174503 |
| HCT116 | Control | ATAC-seq | PE | hg38 | Cho et al.,2018 [4] | GSE97583 |
| HCT116 | Control | Omni ATAC-seq | PE | hg38 | Spektor et al.,2019 [5] | GSE126215 |
| HCT116 | Control | UniNicE-seq | PE | hg38 | Chin et al., 2020(16)* | GSE140276 |
| HCT116 | Control | DNase-seq | SE | hg38 | ENCODE project consortium, 2012 [2] | ENCSR000ENM |
| HCT116 | Control | RNA-seq | PE | hg38 |  | GSE82338 |
| HEK293 | Control | ATAC-seq | PE | hg38 | Calviello et al.,2019 [6] | GSE108513 |
| HEK293 | Control | Omni ATAC-seq | SE | hg38 | Zemke et al., 2019 [7] | GSE130137 |
| HEK293 | Control | RNA-seq | PE | hg38 | Monteys et al., 2021 [8] | GSE176527 |
| GM12878 | 50000_cells | ATAC-seq | PE | hg38 | Buenrostro et al., 2013 (13)* | GSE47753 |
| GM12878 | 500_cells | ATAC-seq | PE | hg38 | Buenrostro et al., 2013 (13)* | GSE47753 |
| GM12878 | Control | Omni ATAC-seq | PE | hg38 | Corces et al., 2017 (15)* | PRJNA380283 |
| GM12878 | Single cell | Single cell ATAC-seq | PE | hg38 | Buenrostro et al., 2015 [9] | GSE65360 |
| GM12878 | Fixed_rep1 | ATAC-see | PE | hg38 | Chen et al., 2016 [10] | GSE76006 |
| GM12878 | Fixed_rep2 | ATAC-see | PE | hg38 | Chen et al., 2016 [10] | GSE76006 |
| GM12878 | Control | RNA-seq | PE | hg38 | Grubert et al., 2020 [11] | GSE134743 |

**References:**

1. Gertz J, Savic D, Varley KE, Partridge EC, Safi A, Jain P, et al. Distinct properties of cell-type-specific and shared transcription factor binding sites. Mol. Cell. 2013; <https://doi.org/10.1016/j.molcel.2013.08.037>
2. Consortium TEP, An integrated encyclopedia of DNA elements in the human genome, Nature. 2012; ﻿10.1038/nature11247
3. Frietze S, Wang R, Yao L, Tak YG, Ye Z, Gaddis M, et al. Cell type-specific binding patterns reveal that TCF7L2 can be tethered to the genome by association with GATA3. Genome biology. 2012; 13(9), R52. <https://doi.org/10.1186/gb-2012-13-9-r52>
4. Cho SW, Xu J, Sun R, Mumbach MR, Carter AC, Chen YG, et al. Promoter of lncRNA Gene PVT1 Is a Tumor-Suppressor DNA Boundary Element, Cell. 2018; <https://doi.org/10.1016/j.cell.2018.03.068>.
5. Spektor R, Tippens ND, Mimoso CA, Soloway PD. methyl-ATAC-seq measures DNA methylation at accessible chromatin. Genome research. 2019; 29(6), 969–977. <https://doi.org/10.1101/gr.245399.118>
6. Karabacak Calviello A, Hirsekorn A, Wurmus R, Yusuf D, Ohler U. Reproducible inference of transcription factor footprints in ATAC-seq and DNase-seq datasets using protocol-specific bias modeling. Genome Biol. 2019; 20, 42. <https://doi.org/10.1186/s13059-019-1654-y>
7. Zemke NR, Gou D, Berk AJ. Dedifferentiation by adenovirus E1A due to inactivation of Hippo pathway effectors YAP and TAZ. Genes & development. 2019; 33(13-14), 828–843. <https://doi.org/10.1101/gad.324814.119>
8. Monteys AM, Hundley AA, Ranum PT, Tecedor L et al. Regulated control of gene therapies by drug-induced splicing. *Nature* 2021 Aug;596(7871):291-295.
9. Buenrostro JD, Wu B, Litzenburger UM, Ruff D, Gonzales ML, Snyder MP, et al. Single-cell chromatin accessibility reveals principles of regulatory variation. Nature. 2015; 523, 486–490. <https://doi.org/10.1038/nature14590>
10. Chen X, Shen Y, Draper W, Buenrostro JD, Litzenburger U, Cho SW, et al. ATAC-see reveals the accessible genome by transposase-mediated imaging and sequencing, Nat. Methods. 2016; <https://doi.org/10.1038/nmeth.4031>.
11. Grubert F, Srivas R, Spacek DV, Kasowski M et al. Landscape of cohesin-mediated chromatin loops in the human genome. *Nature* 2020 Jul;583(7818):737-743.

*These references are cited in main manuscript
